# K_2_CO_3_-Promoted oxy-Michael Addition/Cyclization of *α*,*β*-Unsaturated Carbonyl Compounds with Naphthols: Synthesis of Naphthopyrans

**DOI:** 10.3390/molecules28145502

**Published:** 2023-07-19

**Authors:** Shan-Shan Li, Li-Li Zhao, Min Pan, Na Feng, Jin-Bao Peng, Ai-Jun Ma

**Affiliations:** School of Biotechnology and Health Sciences, Wuyi University, Jiangmen 529020, China; lishanshanlishan@126.com (S.-S.L.); zli_lia2023@163.com (L.-L.Z.); pminl2@163.com (M.P.); wyuchemfn@126.com (N.F.); pengjb_05@126.com (J.-B.P.)

**Keywords:** potassium carbonate, naphthopyrans, tandem oxy-Michael addition/cyclization, synthesis

## Abstract

A potassium carbonate promoted tandem oxy-Michael addition/cyclization of *α*,*β*-unsaturated carbonyl compounds with naphthol derivatives for the synthesis of 2-substituted naphthopyrans was developed. Using the readily available, inexpensive potassium carbonate as the promoter, a range of different substituted naphthopyrans were prepared.

## 1. Introduction

**Figure 1:**
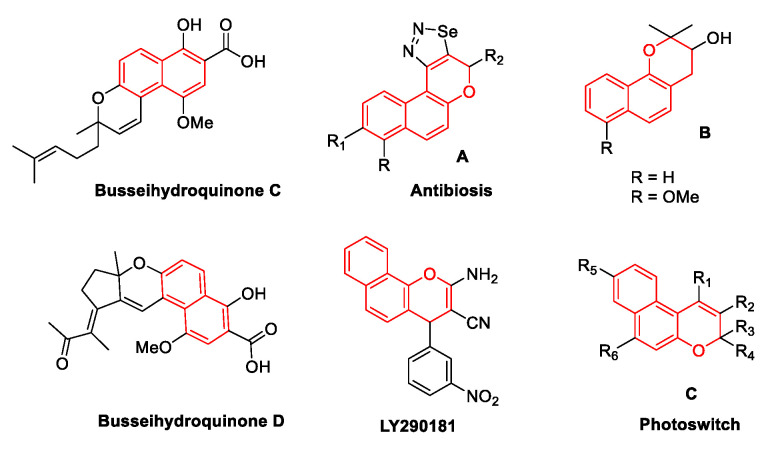
Representative naphthopyrans used in medicine and industry.

Naphthopyran derivatives are widely used in medicine and industry. Many naphthopyran derivatives have important biological and pharmacological activities. Natural products with a naphthopyran core, busseihydroquinones **C**–**D**, have been isolated from the roots of *Pentas bussei*, a plant found in Kenya, and a decoction of the roots is used as a remedy for gonorrhea, syphilis, and dysentery [1,2]. Compound **A** has shown anti-bacterial activity against some gram-positive and gram-negative bacteria [3,4]. Compound **B** showed low cytotoxicity toward KB cells in vitro and was inactive against bacteria and fungi [5,6]. In addition, naphthopyran analogs of LY290181 can act as tumor vascular disrupting agents [7]. Compound **C** with a naphthopyran core exhibits photochromic properties, which are useful for many applications, e.g., in the manufacture of ophthalmic lenses, contact lenses, solar protection glasses, filters, camera optics, transmission devices, agrochemical films, glazes, decorative objects, and in information storage using optical inscription (Figure 1) [5,6,8,9,10,11].

**Scheme 1:**
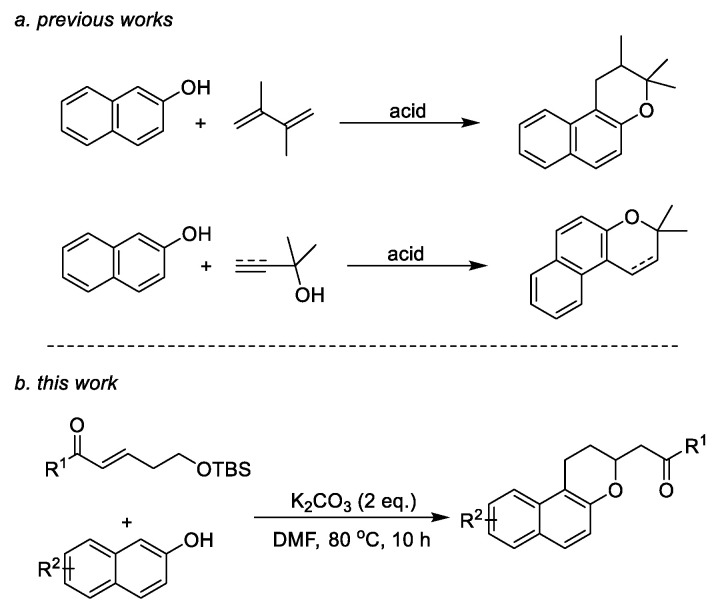
Approaches to naphthopyrans.

Therefore, efficient methods toward the synthesis of the naphthopyran skeleton have long been valued by synthetic chemists, and many synthetic strategies have been reported. The main route involves the reaction of naphthols and olefins catalyzed by a Lewis acid, including the reaction of naphthols with conjugated dienes [4,12,13,14,15] and the reaction of naphthols with analogs, such as allyl alcohol [5,9,16,17,18,19,20,21,22,23,24]. In these strategies, precious-metal catalysts or a large amount of acid catalysts are used. More importantly, this type of reaction is not suitable for the synthesis of acid-sensitive natural products. Therefore, it is desirable to develop a method to obtain naphthopyran compounds using alkali base (Scheme 1a).

We speculated that the reaction sites of phenolic hydroxyl groups and unsaturated ketones can be subjected to a base [25] to obtain naphthopyran products, while the remaining carbonyl group in the product would allow for further structural modification, enabling the synthesis of various pharmacological compounds (Scheme 1b).

## 2. Results and Discussion

Here, we investigated the activity of readily available, inexpensive potassium carbonate as a promoter for the synthesis of naphthopyran compounds by the reaction of naphthol with unsaturated ketones. The results indicated that this method may be an economic, efficient, and practical synthesis for naphthopyran natural products.

In the first set of experiments, we used *tert*-butyldimethylsilyl (TBS) as the protecting group for the screening of subsequent reaction conditions. Different solvents, THF, DMSO, MeOH, 1,4-dioxane, DCE, and EtOAc, were then examined; only low yields were obtained and most of the raw materials did not react (Table 1, entries 1–7). The reaction proceeded efficiently in the strong polar solvent DMF and provided the product **4aa** in 41% yield (Table 1, entry 8). We then examined different bases for the reaction in DMF. However, no desired product **4aa** was obtained when *t*-BuOK or pyridine were used (Table 1, entries 9–10). When CsF or K_3_PO_4_ were used, only a trace amount of product was obtained (Table 1, entries 11–12). K_2_CO_3_ was found to be the optimal base and **4aa** was obtained in an excellent yield of 91% and was successfully isolated in 88% yield (Table 1, entry 13). Raising the temperature to 80 °C increased the reaction rate.

**Scheme 2:**
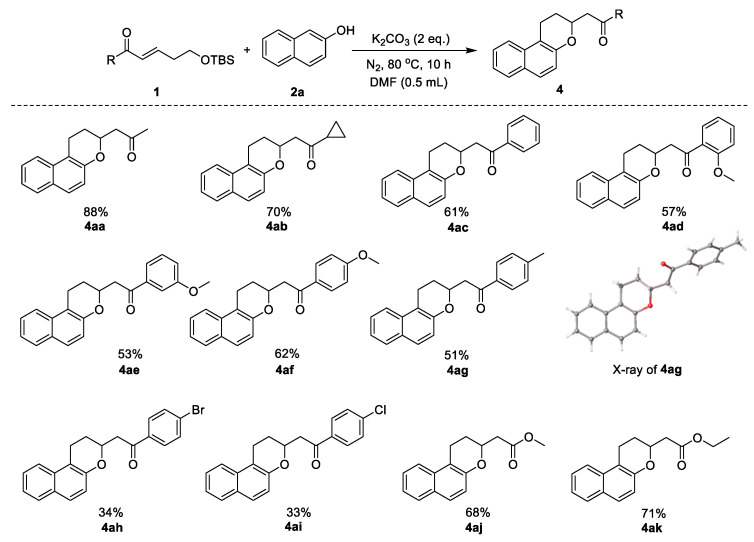
Substrate scope of *α*,*β*-unsaturated carbonyls in the synthesis of 2-substituted naphthopyrans. Reaction conditions: naphthalen-2-ol (1.5 equiv), K_2_CO_3_ (2 equiv), DMF (0.5 mL), 80 °C, 10 h. Isolated yields.

Having established the optimized reaction conditions, we investigated the substrate scope of the reaction with various *α*,*β*-unsaturated carbonyls and naphthalens. As summarized in Scheme 2, we first investigated the reaction of naphthalen-2-ol **2a** with a variety of *α*,*β*-unsaturated carbonyls 1 under the optimal conditions. Substituted α,β-unsaturated carbonyls, with both electron-donating (**4ad**–**4ag**) and electron-withdrawing (**4ah** and **4ai**) groups at different positions on the benzene ring, were suitable substrates and gave the desired 2-substituted naphthopyran products. The structure of **4ag** was unambiguously determined by single-crystal X-ray diffraction analysis (CCDC 2171562 (**4ag**) contains the supplementary crystallographic data for this paper. For details, see the supporting information). A cyclopropane-substituted, α,β-unsaturated carbonyl compound was also tolerated, giving the corresponding product in 70% yield (**4ab**). In addition, esters could also react with naphthol and gave the naphthopyran products (**4aj**–**4ak**) in good yields.

**Scheme 3:**
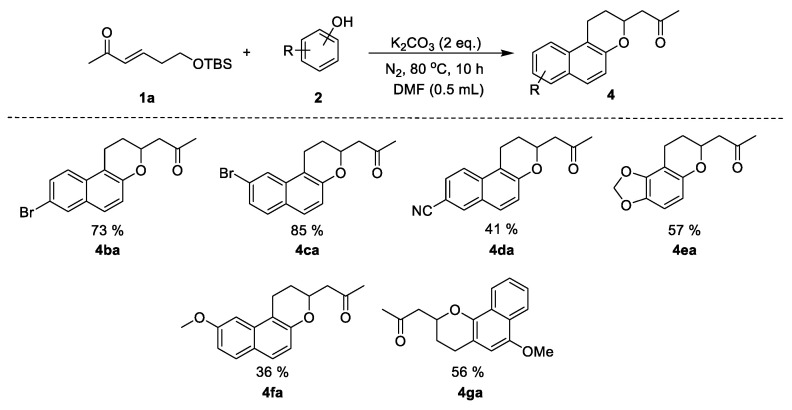
Substrate scope of naphthalens in the synthesis of 2-substituted naphthopyrans. Reaction conditions: naphthalen-2-ol (1.5 equiv), K_2_CO_3_ (2 equiv), DMF (0.5 mL), 80 °C, 10 h. Isolated yields.

**Scheme 4:**
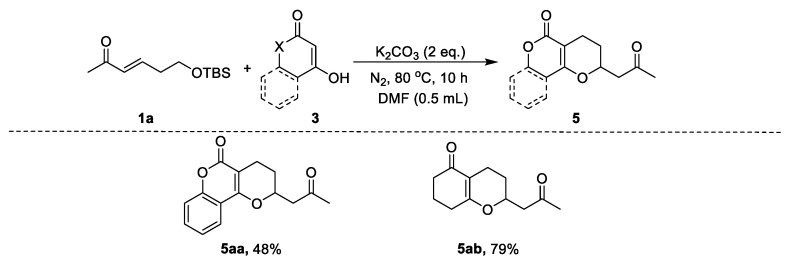
Substrate scope of 1,3-dicarbonyl compounds in the synthesis of pyrans. Reaction conditions: naphthalen-2-ol (1.5 equiv), K_2_CO_3_ (2 equiv), DMF (0.5 mL), 80 °C, 10 h. Isolated yields.

We further investigated the scope of the naphthalens for this reaction. As illustrated in Scheme 3, a series of different naphthalens **2** were reacted with *α*,*β*-unsaturated carbonyl **1a** under the optimized conditions. This transformation showed good generality and reactivity; all the tested naphthalens were tolerated and produced the corresponding 2-substituted naphthopyran **4**. Starting materials bearing electron-withdrawing and electron-donating groups afforded the corresponding products (Scheme 3, **4ba**–**4ga**). Moreover, 4-hydroxycoumarin and 1,3-cyclohexanedione were also tolerated and provided the desired products in 48% and 79% yield, respectively (Scheme 4, **5aa** and **5ab**).

**Scheme 5:**
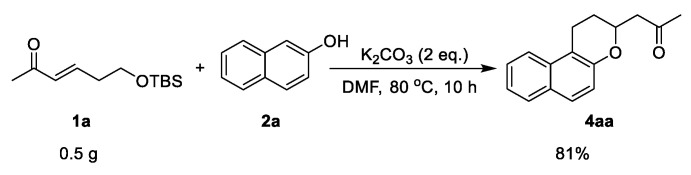
The gram-scale reaction.

Subsequently, we explored a gram-scale reaction. *α*,*β*-Unsaturated carbonyl **1a** was treated with naphthalen-2-ol **2a** under the standard conditions (Scheme 5). The corresponding naphthopyran product **4aa** was obtained in 81% yield, slightly lower than the small-scale reaction yield.

**Scheme 6:**
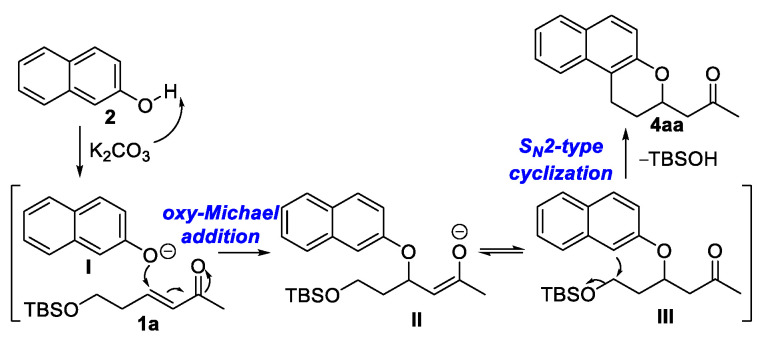
Possible mechanism for the formation of **4aa**.

The reaction of *α*,*β*-Unsaturated carbonyl **1a** and 1-naphthol was checked as well, which produced the desired product in 31% yield (See NMR spectra in Supplementary Materials). Herein, we proposed an immature process. Initially, deprotonation of the OH group of 2-naphthol **2a** with K_2_CO_3_ generated 2-naphtholanion **I**, which undergoes an oxy-Michael addition reaction to the *α*,*β*-unsaturated carbonyl compound **1** to afford the alkylated naphthol intermediate **III**. This intermediate then might undergo an intramolecular S_N_2-type cyclization process to form the 2-substituted naphthopyran product **4aa** along with the loss of TBSOH (Scheme 6). However, we have to realize that the mechanism of the reaction is unclear at this stage.

In summary, we have developed an approach to access 2-substituted naphthopyrans using *α*,*β*-unsaturated carbonyl compounds and naphthol derivatives in DMF at 80 °C with K_2_CO_3_ as the promoter. The reaction is proposed to undergo a tandem oxy-Michael addition/cyclization process, and various substitutions on both reaction partners were tolerated. This reaction is the first method for synthesizing naphthopyran compounds using basic medium.

## 3. Materials and Methods

### 3.1. General Information

Unless otherwise noted, all reactions were carried out under air atmosphere. 1,2-dichloroethane, N,N-Dimethylformamide, naphthols, ferric chloride, and potassium carbonate were from commercial sources and used as received without further purification. Reactions were monitored by thin-layer chromatography (TLC) carried out on 0.20 mm silica gel plates using UV light as the visualizing agent, and iodine and an acidic solution of phosphomolybdic acid (PMA) with heat as the stains. Column chromatography was performed on silica gel (200–300 meshes) using petroleum ether and ethyl acetate as eluent. All new compounds were characterized by means of ^1^H NMR, ^13^C NMR and HRMS. NMR spectra were recorded using a Bruker AVANCE NEO 500 MHz NMR spectrometer and can be found at the end of the paper. All ^1^HNMR data are reported in δ units, parts per million (ppm), and were calibrated relative to the signals for residual chloroform (7.26 ppm) in deuterochloroform (CDCl_3_). All ^13^C NMR data are reported in ppm relative to CDCl_3_ (77.16 ppm) and were obtained with ^1^H decoupling. The following abbreviations or combinations, thereof, were used to explain the multiplicities: s = singlet, bs = broad singlet, d = doublet, t = triplet, q = quartet, m = multiplet. Single crystal X-ray diffraction (SCXRD) of **4ag** was carried out at 100(2) K on a Bruker D8 VENTURE diffractometer using Mo-Kα radiation (λ = 0.71073 Å). Integration and scaling of intensity data was performed using the SAINT program. Data were corrected for the effects of absorption using SADABS. The structures were solved by direct method and refined with the full-matrix least-squares technique using SHELX-2014 software. Non-hydrogen atoms were refined with anisotropic displacement parameters, and hydrogen atoms were placed in calculated positions and refined with a riding model.

### 3.2. General Information

*α*,*β*-Unsaturated carbonyl compound **1** (0.2 mmol, 1.0 equiv), naphthol **2** (0.3 mmol, 1.5 equiv), and K_2_CO_3_ (0.4 mmol, 2.0 equiv) were added into a 15 mL tube under N_2_ atmosphere. DMF (1.5 mL) was added to the reaction tube. Then, the reaction mixture was heated to 80 °C. After a time period of 10 h, the solution was diluted with EtOAc (2 mL × 3), washed with brine (1 mL × 1), and concentrated in vacuo. The crude product was purified by a flash chromatography on silica gel to afford the corresponding product **4**.

### 3.3. Characterization of Products in Details

*1-(2,3-dihydro-1H-benzo[f]chromen-3-yl)propan-2-one* (**4aa**):

The title compound was prepared from (*E*)-6-((tert-butyldimethylsilyl)oxy)hex-3-en-2-one (0.2 mmol), naphthalen-2-ol (0.3 mmol), and potassium carbonate (0.4 mmol). The product was a colorless oil. ^1^H NMR (500 MHz, CDCl_3_) δ 8.09–8.05 (m, 1H), 7.75–7.72 (m, 1H), 7.44–7.40 (m, 2H), 7.34 (d, *J* = 8.3 Hz, 1H), 7.15 (d, *J* = 8.4 Hz, 1H), 4.69–4.62 (m, 1H), 3.13–2.97 (m, 2H), 2.88–2.83 (m, 1H), 2.77 (dd, *J* = 15.8, 5.3 Hz, 1H), 2.34 (s, 3H), 2.22–2.15 (m, 1H), 1.92–1.82 (m, 1H). ^13^C NMR (126 MHz, CDCl_3_) δ 206.7, 149.0, 133.1, 127.6, 127.4, 125.6, 125.2, 125.1, 121.2, 119.8, 115.3, 72.5, 49.1, 31.1, 27.4, 24.6. HRMS (ESI) *m*/*z* 263.1053 [(M+Na)^+^; calcd for C_16_H_16_O_2_Na: 263.1048].

*1-cyclopropyl-2-(2,3-dihydro-1H-benzo[f]chromen-3-yl)ethan-1-one* (**4ab**):

The title compound was prepared from (*E*)-5-((tert-butyldimethylsilyl)oxy)-1-cyclopropylpent-2-en-1-one (0.2 mmol), naphthalen-2-ol (0.3 mmol), and potassium carbonate (0.4 mmol). The product was a white solid. ^1^H NMR (500 MHz, CDCl3) δ 7.82 (m, 2H), 7.65 (d, *J* = 8.9 Hz, 1H), 7.53 (m, 1H), 7.39 (m, 1H), 7.07 (d, *J* = 8.9 Hz, 1H), 4.64 (m, 1H), 3.20–3.05 (m, 3H), 2.88 (dd, *J* = 16.0, 6.2 Hz, 1H), 2.30 (m, 1H), 2.07 (m, 1H), 1.92 (m, 1H), 1.25–1.10 (m, 2H), 1.05–0.90 (m, 2H). ^13^C NMR (126 MHz, CDCl_3_) δ 208.4, 152.1, 133.1, 129.0, 128.4, 127.7, 126.4, 123.3, 121.9, 119.1, 113.5, 71.8, 48.5, 27.2, 21.3, 21.0, 11.1, 11.0. HRMS (ESI) *m*/*z* 289.1263 [(M+Na)^+^; calcd for C_18_H_18_O_2_Na: 289.1199].

*2-(2,3-dihydro-1H-benzo[f]chromen-3-yl)-1-phenylethan-1-one* (**4ac**):

The title compound was prepared from (*E*)-5-((tert-butyldimethylsilyl)oxy)-1-phenylpent-2-en-1-one (0.2 mmol), naphthalen-2-ol (0.3 mmol), and potassium carbonate (0.4 mmol). The product was a yellow solid. ^1^H NMR (500 MHz, CDCl_3_) δ 8.05 (d, *J* = 8.3 Hz, 2H), 7.84 (d, *J* = 8.5 Hz, 1H), 7.78 (d, *J* = 9.8 Hz, 1H), 7.65–7.59 (m, 2H), 7.52 (t, *J* = 8.1 Hz, 3H), 7.40–7.35 (m, 1H), 7.03 (d, *J* = 8.9 Hz, 1H), 4.80 (m, 1H), 3.66 (dd, *J* = 16.4, 6.0 Hz, 1H), 3.27 (dd, *J* = 16.4, 6.7 Hz, 1H), 3.17 (m, 2H), 2.42 (m, 1H), 1.99 (m, 1H). ^13^C NMR (126 MHz, CDCl_3_) δ 197.7, 152.1, 137.1, 133.4, 133.0, 129.0, 128.7, 128.4, 128.3, 127.7, 126.4, 123.3, 121.9, 119.1, 113.5, 72.1, 43.9, 27.3, 21.0. HRMS (ESI) *m*/*z* 303.1389 [(M+H)^+^; calcd for C_21_H_19_O_2_: 303.1385].

*2-(2,3-dihydro-1H-benzo[f]chromen-3-yl)-1-(2-methoxyphenyl)ethan-1-one* (**4ad**):

The title compound was prepared from (*E*)-5-((tert-butyldimethylsilyl)oxy)-1-(2-metho-xyphenyl)pent-2-en-1-one (0.2 mmol), naphthalen-2-ol (0.3 mmol), and potassium carbonate (0.4 mmol). The product was a yellow solid. ^1^H NMR (500 MHz, CDCl_3_) δ 7.84 (d, *J* = 9.5 Hz, 1H), 7.79 (m, 2H), 7.62 (d, *J* = 8.8 Hz, 1H), 7.51 (m, 2H), 7.37 (t, *J* = 7.5 Hz, 1H), 7.10–6.97 (m, 3H), 4.76 (m, 1H), 3.93 (s, 3H), 3.64 (dd, *J* = 16.8, 6.2 Hz, 1H), 3.37 (dd, *J* = 16.8, 6.8 Hz, 1H), 3.19–3.07 (m, 2H), 2.38 (m, 1H), 1.96 (m, 1H). ^13^C NMR (126 MHz, CDCl_3_) δ 199.7, 158.7, 152.3, 133.7, 133.1, 130.4, 128.9, 128.4, 128.3, 127.6, 126.3, 123.2, 121.9, 120.8, 119.2, 113.6, 111.6, 72.2, 55.6, 49.1, 27.2, 21.1. HRMS (ESI) *m*/*z* 333.1492 [(M+H)^+^; calcd for C_22_H_21_O_3_: 333.1491].

*2-(2,3-dihydro-1H-benzo[f]chromen-3-yl)-1-(3-methoxyphenyl)ethan-1-one* (**4ae**):

The title compound was prepared from (*E*)-5-((tert-butyldimethylsilyl)oxy)-1-(3-methox-yphenyl)pent-2-en-1-one (0.2 mmol), naphthalen-2-ol (0.3 mmol), and potassium carbonate (0.4 mmol). The product was a yellow solid. ^1^H NMR (500 MHz, CDCl_3_) δ 7.84 (d, *J* = 7.3 Hz, 1H), 7.79 (d, *J* = 7.9 Hz, 1H), 7.63 (d, *J* = 9.3 Hz, 2H), 7.58 (s, 1H), 7.52 (m, 1H), 7.45–7.35 (m, 2H), 7.17 (dd, *J* = 8.2, 2.7 Hz, 1H), 7.03 (d, *J* = 8.9 Hz, 1H), 4.80 (m, 1H), 3.90 (s, 3H), 3.64 (dd, *J* = 16.5, 6.1 Hz, 1H), 3.26 (dd, *J* = 16.4, 6.7 Hz, 1H), 3.22–3.08 (m, 2H), 2.41 (m, 1H), 1.99 (m, 1H). ^13^C NMR (126 MHz, CDCl_3_) δ 197.5, 159.9, 152.1, 138.5, 133.0, 129.7, 129.0, 128.4, 127.7, 126.4, 123.3, 121.9, 121.0, 119.9, 119.1, 113.5, 112.4, 72.1, 55.5, 44.0, 27.3, 21.0. HRMS (ESI) *m*/*z* 333.1494 [(M+H)^+^; calcd for C_22_H_21_O_3_: 333.1491].

*2-(2,3-dihydro-1H-benzo[f]chromen-3-yl)-1-(4-methoxyphenyl)ethan-1-one* (**4af**):

The title compound was prepared from (*E*)-5-((tert-butyldimethylsilyl)oxy)-1-(4-methoxy-phenyl)pent-2-en-1-one(0.2 mmol), naphthalen-2-ol (0.3 mmol), and potassium carbonate (0.4 mmol). The product was a yellow solid. ^1^H NMR (500 MHz, CDCl_3_) δ 8.03 (d, *J* = 8.9 Hz, 2H), 7.84 (d, *J* = 8.4 Hz, 1H), 7.78 (d, *J* = 8.1 Hz, 1H), 7.63 (d, *J* = 8.9 Hz, 1H), 7.51 (t, *J* = 8.3 Hz, 1H), 7.37 (t, *J* = 8.0 Hz, 1H), 7.03 (d, *J* = 8.9 Hz, 1H), 6.98 (d, *J* = 8.9 Hz, 2H), 4.83–4.73 (m, 1H), 3.91 (s, 3H), 3.61 (dd, *J* = 16.2, 5.9 Hz, 1H), 3.21 (dd, *J* = 16.2, 6.8 Hz, 1H), 3.18–3.07 (m, 2H), 2.41 (m, 1H), 1.98 (m, 1H). ^13^C NMR (126 MHz, CDCl_3_) δ 196.23, 163.72, 152.13, 133.05, 130.63, 130.25, 128.96, 128.41, 127.66, 126.33, 123.26, 121.95, 119.13, 113.81, 113.56, 72.34, 55.53, 43.52, 27.28, 21.02. HRMS (ESI) *m*/*z* 355.1308 [(M+Na)^+^; calcd for C_22_H_20_O_3_Na: 355.1310].

*2-(2,3-dihydro-1H-benzo[f]chromen-3-yl)-1-(p-tolyl)ethan-1-one* (**4ag**):

The title compound was prepared from (*E*)-5-((tert-butyldimethylsilyl)oxy)-1-(p-tolyl)pent-2-en-1-one(0.2 mmol), naphthalen-2-ol (0.3 mmol), and potassium carbonate (0.4 mmol). The product was a yellow solid. ^1^H NMR (500 MHz, CDCl_3_) δ 7.95 (d, *J* = 8.0 Hz, 2H), 7.84 (d, *J* = 8.4 Hz, 1H), 7.79 (d, *J* = 8.1 Hz, 1H), 7.63 (d, *J* = 8.9 Hz, 1H), 7.52 (t, *J* = 7.7 Hz, 1H), 7.38 (t, *J* = 7.5 Hz, 1H), 7.31 (d, *J* = 8.0 Hz, 2H), 7.04 (d, *J* = 8.9 Hz, 1H), 4.80 (m, 1H), 3.63 (dd, *J* = 16.3, 5.9 Hz, 1H), 3.24 (dd, *J* = 16.3, 6.8 Hz, 1H), 3.16 (m, 2H), 2.52–2.37 (m, 4H), 1.98 (m, 1H). ^13^C NMR (126 MHz, CDCl_3_) δ 197.3, 152.1, 144.2, 134.7, 133.1, 129.4, 129.0, 128.4, 128.4, 127.7, 126.3, 123.3, 122.0, 119.1, 113.5, 72.2, 43.8, 27.3, 21.7, 21.0. HRMS (ESI) *m*/*z* 317.1545 [(M+H)^+^; calcd for C_22_H_21_O_2_: 317.1542].

*1-(4-bromophenyl)-2-(2,3-dihydro-1H-benzo[f]chromen-3-yl)ethan-1-one* (**4ah**):

The title compound was prepared from (*E*)-1-(4-bromophenyl)-5-((tert-butyldimethylsilyl)oxy)-pent-2-en-1-one (0.2 mmol), naphthalen-2-ol (0.3 mmol), and potassium carbonate (0.4 mmol). The product as a colorless oil. ^1^H NMR (500 MHz, CDCl_3_) δ 7.91 (d, *J* = 8.6 Hz, 2H), 7.84 (d, *J* = 8.5 Hz, 1H), 7.78 (d, *J* = 8.1 Hz, 1H), 7.68–7.60 (m, 3H), 7.54–7.49 (m, 1H), 7.40–7.33 (m, 1H), 7.00 (d, *J* = 8.9 Hz, 1H), 4.77 (m, 1H), 3.61 (dd, *J* = 16.3, 6.3 Hz, 1H), 3.24–3.11 (m, 3H), 2.40 (m, 1H), 1.99 (m, 1H). ^13^C NMR (126 MHz, CDCl_3_) δ 196.83, 151.94, 135.78, 132.98, 132.01, 129.87, 128.98, 128.64, 128.44, 127.75, 126.41, 123.37, 121.94, 119.02, 113.47, 72.06, 43.80, 27.25, 20.99. HRMS (ESI) *m*/*z* 403.0312 [(M+Na)^+^; calcd for C_21_H_17_O_2_NaBr: 403.0310].

*1-(4-chlorophenyl)-2-(2,3-dihydro-1H-benzo[f]chromen-3-yl)ethan-1-one* (**4ai**):

The title compound was prepared from (*E*)-5-((tert-butyldimethylsilyl)oxy)-1-(4-chlorophenyl)-pent-2-en-1-one(0.2 mmol), naphthalen-2-ol (0.3 mmol), and potassium carbonate (0.4 mmol). The product was a colorless oil. ^1^H NMR (500 MHz, CDCl_3_) δ 7.99 (d, *J* = 8.6 Hz, 2H), 7.84 (d, *J* = 8.4 Hz, 1H), 7.78 (d, *J* = 8.1 Hz, 1H), 7.62 (d, *J* = 8.9 Hz, 1H), 7.54–7.46 (m, 3H), 7.38 (t, *J* = 7.5 Hz, 1H), 7.00 (d, *J* = 8.9 Hz, 1H), 4.78 (m, 1H), 3.62 (dd, *J* = 16.3, 6.3 Hz, 1H), 3.21 (dd, *J* = 16.4, 6.3 Hz, 1H), 3.18–3.07 (m, 2H), 2.44–2.36 (m, 1H), 1.99 (m, 1H). ^13^C NMR (126 MHz, CDCl_3_) δ 196.62, 151.95, 139.87, 135.39, 132.98, 129.76, 129.01, 128.98, 128.44, 127.75, 126.40, 123.36, 121.93, 119.03, 113.47, 72.07, 43.82, 27.26, 21.00. HRMS (ESI) *m*/*z* 359.0807 [(M+Na)^+^; calcd for C_21_H_17_O_2_NaCl: 359.0815].

*methyl 2-(2,3-dihydro-1H-benzo[f]chromen-3-yl)acetate* (**4aj**):

The title compound was prepared from methyl (*E*)-5-((tert-butyldimethylsilyl)oxy)pent-2-enoate (0.2 mmol), naphthalen-2-ol (0.3 mmol), and potassium carbonate (0.4 mmol). The product was a yellow solid. ^1^H NMR (500 MHz, Chloroform-d) δ 7.83–7.74 (m, 2H), 7.62 (d, *J* = 8.8 Hz, 1H), 7.49 (m, 1H), 7.35 (m, 1H), 7.04 (d, *J* = 8.9 Hz, 1H), 4.59–4.53 (m, 1H), 3.77 (d, *J* = 1.8 Hz, 3H), 3.17–3.03 (m, 2H), 2.88 (dd, *J* = 15.5, 7.3 Hz, 1H), 2.69 (dd, *J* = 15.5, 6.0 Hz, 1H), 2.32–2.24 (m, 1H), 1.98–1.89 (m, 1H). ^13^C NMR (126 MHz, CDCl_3_) δ 171.22, 151.87, 132.90, 128.94, 128.37, 127.68, 126.30, 123.27, 121.84, 119.05, 113.25, 71.94, 51.83, 40.09, 26.92, 20.93. HRMS (ESI) *m*/*z* 279.0999 [(M+Na)^+^; calcd for C_16_H_16_O_3_Na: 279.0997].

*methyl 2-(2,3-dihydro-1H-benzo[f]chromen-3-yl)acetate* (**4ak**):

The title compound was prepared from ethyl (*E*)-5-((tert-butyldimethylsilyl)oxy)pent-2-enoate (0.2 mmol), naphthalen-2-ol (0.3 mmol), and potassium carbonate (0.4 mmol). The product was a yellow solid. ^1^H NMR (500 MHz, Chloroform-d) δ 7.81 (dd, *J* = 11.7, 8.3 Hz, 2H), 7.64 (d, *J* = 8.9 Hz, 1H), 7.51 (t, *J* = 7.3 Hz, 1H), 7.37 (t, *J* = 7.4 Hz, 1H), 7.06 (d, *J* = 8.9 Hz, 1H), 4.59–4.57 (m, 1H), 4.25 (q, *J* = 7.1 Hz, 2H), 3.22–3.03 (m, 2H), 2.89 (dd, *J* = 15.3, 7.3 Hz, 1H), 2.69 (dd, *J* = 15.3, 6.0 Hz, 1H), 2.33–2.29 (m, 1H), 1.98–1.94 (m, 1H), 1.34 (t, *J* = 7.1 Hz, 3H). ^13^C NMR (126 MHz, CDCl_3_) δ 170.84, 151.98, 132.97, 128.99, 128.43, 127.72, 126.35, 123.31, 121.90, 119.12, 113.33, 72.09, 60.73, 40.41, 26.99, 21.00, 14.28. HRMS (ESI) *m*/*z* 293.1153 [(M+Na)^+^; calcd for C_17_H_18_O_3_Na: 293.1154].

*1-(8-bromo-2,3-dihydro-1H-benzo[f]chromen-3-yl)propan-2-one* (**4ba**):

The title compound was prepared from (*E*)-6-((tert-butyldimethylsilyl)oxy)hex-3-en-2-one (0.2 mmol), 6-bromonaphthalen-2-ol (0.3 mmol), and potassium carbonate (0.4 mmol). The product was a white solid. ^1^H NMR (500 MHz, CDCl_3_) δ 7.90 (d, *J* = 2.1 Hz, 1H), 7.65 (d, *J* = 8.9 Hz, 1H), 7.55 (dd, *J* = 9.0, 2.1 Hz, 1H), 7.51 (d, *J* = 8.9 Hz, 1H), 7.04 (d, *J* = 9.0 Hz, 1H), 4.61–4.51 (m, 1H), 3.13–2.97 (m, 3H), 2.73 (dd, *J* = 16.3, 5.5 Hz, 1H), 2.33–2.22 (m, 4H), 1.87 (m, 1H). ^13^C NMR (126 MHz, CDCl_3_) δ 206.3, 152.3, 131.6, 130.3, 130.2, 129.5, 126.8, 123.8, 120.2, 117.0, 113.7, 71.8, 48.8, 31.0, 27.0, 21.0. HRMS (ESI) *m*/*z* 319.0337 [(M+H)^+^; calcd for C_16_H_16_BrO_2_: 319.0334].

*1-(9-bromo-2,3-dihydro-1H-benzo[f]chromen-3-yl)propan-2-one* (**4ca**):

The title compound was prepared from (*E*)-6-((tert-butyldimethylsilyl)oxy)hex-3-en-2-one (0.2 mmol), 7-bromonaphthalen-2-ol (0.3 mmol), and potassium carbonate (0.4 mmol). The product was a white solid. ^1^H NMR (500 MHz, CDCl_3_) δ 7.95 (d, *J* = 1.9 Hz, 1H), 7.62 (d, *J* = 8.6 Hz, 1H), 7.57 (d, *J* = 8.8 Hz, 1H), 7.43 (dd, *J* = 8.6, 1.9 Hz, 1H), 7.02 (d, *J* = 8.9 Hz, 1H), 4.62–4.48 (m, 1H), 3.09–2.95 (m, 3H), 2.73 (dd, *J* = 16.3, 5.4 Hz, 1H), 2.33–2.21 (m, 4H), 1.87 (m, 1H). ^13^C NMR (126 MHz, CDCl_3_) δ 206.3, 152.8, 134.4, 130.0, 127.6, 127.4, 126.6, 124.4, 120.9, 119.5, 112.8, 71.9, 48.8, 31.0, 26.9, 21.0. HRMS (ESI) *m*/*z* 319.0336 [(M+H)^+^; calcd for C_16_H_16_BrO_2_: 319.0334].

*3-(2-oxopropyl)-2,3-dihydro-1H-benzo[f]chromene-8-carbonitrile* (**4da**):

The title compound was prepared from (*E*)-6-((tert-butyldimethylsilyl)oxy)hex-3-en-2-one (0.2 mmol), 6-hydroxy-2-naphthonitrile (0.3 mmol), and potassium carbonate (0.4 mmol). The product was a colorless oil. ^1^H NMR (500 MHz, CDCl_3_) δ 8.10 (d, *J* = 1.7 Hz, 1H), 7.84 (d, *J* = 8.8 Hz, 1H), 7.65–7.56 (m, 2H), 7.11 (d, *J* = 9.0 Hz, 1H), 4.63–4.57 (m, 1H), 3.10–3.00 (m, 3H), 2.76 (dd, *J* = 16.5, 5.5 Hz, 1H), 2.29 (s, 4H), 1.88 (m, 1H). ^13^C NMR (126 MHz, CDCl_3_) δ 206.0, 154.6, 134.8, 134.3, 128.2, 127.9, 127.1, 123.1, 121.0, 119.6, 113.9, 106.4, 72.1, 48.6, 31.0, 26.7, 21.0. HRMS (ESI) *m*/*z* 266.1177 [(M+H)^+^; calcd for C_17_H_16_NO_2_: 266.1181].

*1-(8,9-dihydro-7H-*[1,3]*dioxolo[4,5-f]chromen-7-yl)propan-2-one* (**4ea**):

The title compound was prepared from (*E*)-6-((tert-butyldimethylsilyl)oxy)hex-3-en-2-one (0.2 mmol), benzo[d][1,3]dioxol-5-ol (0.3 mmol), and potassium carbonate (0.4 mmol). The product was a colorless oil. ^1^H NMR (500 MHz, CDCl_3_) δ 6.50 (s, 1H), 6.33 (s, 1H), 5.87 (q, *J* = 1.4 Hz, 2H), 4.47–4.34 (m, 1H), 2.92 (dd, *J* = 16.1, 7.3 Hz, 1H), 2.81 (m, 1H), 1.78–1.66 (m, 1H). ^13^C NMR (126 MHz, CDCl_3_) δ 206.5, 148.9, 146.4, 141.4, 113.0, 108.2, 100.8, 98.6, 72.0, 49.0, 31.0, 27.4, 24.5. HRMS (ESI) *m*/*z* 235.0972 [(M+H)^+^; calcd for C_13_H_15_O_4_: 235.0970].

*1-(9-methoxy-2,3-dihydro-1H-benzo[f]chromen-3-yl)propan-2-one* (**4fa**):

The title compound was prepared from (*E*)-6-((tert-butyldimethylsilyl)oxy)hex-3-en-2-one (0.2 mmol), 7-methoxynaphthalen-2-ol (0.3 mmol), and potassium carbonate (0.4 mmol). The product was a colorless oil. ^1^H NMR (500 MHz, CDCl_3_) δ 7.68 (d, *J* = 8.8 Hz, 1H), 7.56 (d, *J* = 8.8 Hz, 1H), 7.08 (d, *J* = 2.5 Hz, 1H), 7.04 (dd, *J* = 8.8, 2.5 Hz, 1H), 6.90 (d, *J* = 8.8 Hz, 1H), 4.61–4.53 (m, 1H), 3.95 (s, 3H), 3.07–2.99 (m, 3H), 2.74 (dd, *J* = 16.1, 5.6 Hz, 1H), 2.32–2.24 (m, 4H), 1.96–1.85 (m, 1H). ^13^C NMR (126 MHz, CDCl_3_) δ 206.6, 158.3, 152.6, 134.3, 130.0, 127.5, 124.2, 116.5, 115.1, 112.5, 101.4, 71.7, 55.3, 48.8, 31.1, 27.2, 21.2. HRMS (ESI) *m*/*z* 271.1339 [(M+H)^+^; calcd for C_17_H_19_O_3_: 271.1334].

*1-(6-methoxy-3,4-dihydro-2H-benzo[h]chromen-2-yl)propan-2-one* (**4ga**):

The title compound was prepared from (*E*)-6-((tert-butyldimethylsilyl)oxy)hex-3-en-2-one (0.2 mmol), 4-methoxynaphthalen-1-ol (0.3 mmol), and potassium carbonate (0.4 mmol). The product was a colorless oil. ^1^H NMR (500 MHz, CDCl_3_) δ 8.24–8.13 (m, 1H), 8.13–8.00 (m, 1H), 7.48 (m, 2H), 6.49 (s, 1H), 4.60 (m, 1H), 3.96 (s, 3H), 3.19–2.90 (m, 2H), 2.90–2.67 (m, 2H), 2.34 (s, 3H), 2.17 (m, 1H), 1.94–1.79 (m, 1H). ^13^C NMR (126 MHz, CDCl_3_) δ 206.9, 149.1, 143.0, 126.0, 125.9, 125.1, 125.1, 121.7, 121.0, 114.6, 105.1, 72.3, 55.7, 49.2, 31.1, 27.7, 25.0. HRMS (ESI) *m*/*z* 293.1149 [(M+Na)^+^; calcd for C_17_H_18_O_3_Na: 293.1154].

*2-(2-oxopropyl)-3,4-dihydro-2H,5H-pyrano[3,2-c]chromen-5-one* (**5aa**):

The title compound was prepared from (*E*)-6-((tert-butyldimethylsilyl)oxy)hex-3-en-2-one (0.2 mmol), 4-hydroxy-2H-chromen-2-one (0.3 mmol), and potassium carbonate (0.4 mmol). The product was a colorless oil. ^1^H NMR (500 MHz, CDCl_3_) δ 7.68 (dd, *J* = 7.9, 1.6 Hz, 1H), 7.51 (ddd, *J* = 8.6, 7.3, 1.6 Hz, 1H), 7.32 (dd, *J* = 8.4, 1.1 Hz, 1H), 7.26 (ddd, *J* = 8.2, 7.4, 1.1 Hz, 1H), 4.75 (m, 1H), 3.10 (dd, *J* = 16.9, 7.4 Hz, 1H), 2.81 (dd, *J* = 16.9, 5.3 Hz, 1H), 2.70 (m, 1H), 2.60 (m, 1H), 2.32 (s, 3H), 2.22 (m, 1H), 1.80 (m, 1H). ^13^C NMR (126 MHz, CDCl_3_) δ 204.9, 163.0, 159.5, 152.4, 131.5, 123.8, 122.2, 116.6, 115.6, 101.1, 73.7, 48.2, 31.0, 26.1, 18.9. HRMS (ESI) *m*/*z* 281.0795 [(M+Na)^+^; calcd for C_15_H_14_O_4_Na: 281.0790].

*1-(2-oxopropyl)-2,3,4,6,7,8-hexahydro-5H-chromen-5-one* (**5ab**):

The title compound was prepared from (*E*)-6-((tert-butyldimethylsilyl)oxy)hex-3-en-2-one (0.2 mmol), 3-hydroxycyclohex-2-en-1-one (0.3 mmol), and potassium carbonate (0.4 mmol). The product was a white solid. ^1^H NMR (500 MHz, CDCl_3_) δ 4.45–4.38 (m, 1H), 2.86 (dd, *J* = 16.7, 7.6 Hz, 1H), 2.60 (dd, *J* = 16.7, 5.1 Hz, 1H), 2.40–2.30 (m, 5H), 2.21 (s, 3H), 2.19–2.10 (m, 1H), 2.00–1.86 (m, 3H), 1.58–1.49 (m, 1H). ^13^C NMR (126 MHz, CDCl_3_) δ 205.5, 198.2, 170.9, 111.3, 73.1, 48.3, 36.6, 30.8, 28.5, 26.5, 20.8, 17.3. HRMS (ESI) *m*/*z* 231.0995 [(M+Na)^+^; calcd for C_12_H_16_O_3_Na: 231.0997].

*The gram-scale reaction*:

(*E*)-6-((tert-butyldimethylsilyl)oxy)hex-3-en-2-one (0.2 mmol), naphthalen-2-ol (0.3 mmol), and potassium carbonate (0.4 mmol) were added into a round bottom flask under N_2_ atmosphere. N,N-Dimethylformamide was added to the reaction tube. Then, the reaction mixture was heated to 80 °C. After a time period of 10 h, the solution was diluted with ethyl acetate, washed with brine, and concentrated in vacuo. The crude product was purified by column chromatography on silica gel, eluting with EtOAc/petroleum ether (1/20, *v*/*v*) to afford the corresponding product.

## Figures and Tables

**Table 1:** Optimization of the reaction conditions (a).

Entry	Solvent	Base	Yield (%) (b)
1	THF	DBU	26
2	DMSO	DBU	17
3	MeOH	DBU	37
4	EtOAc	DBU	20
5	1,4-Dioxane	DBU	32
6	Toluene	DBU	27
7	DCE	DBU	28
8	DMF	DBU	**41**
9	DMF	*t*-BuOK	0
10	DMF	pyridine	0
11	DMF	CsF	17
12	DMF	K_3_PO_4_	9
13	DMF	K_2_CO_3_	**91**(88) (c)

(a). Standard conditions: naphthalen-2-ol (1.5 equiv), base (2 equiv), solvent (0.5 mL), 80 °C, 10 h. (b). GC yield, N-dodecane was used as an internal standard. (c). Isolated yield.

## Data Availability

The data for the synthesized compounds presented in this study are available in the Supplementary Materials.

## Supplementary Materials

The following supporting information can be downloaded at: https://www.mdpi.com/article/10.3390/molecules28145502/s1, Table S1: Optimization of Protecting groups; Table S2: Optimization of Solvents; Table S3: Optimization of Bases; X-ray data for products **4ag**; copies of ^1^H, ^13^C spectra. References [26,27,28,29] are cited in the Supplementary Materials.

## Sample Availability

Not available.
