# Supplementary material for: K2CO3-Promoted oxy-Michael Addition/Cyclization of α,β-Unsaturated Carbonyl Compounds with Naphthols: Synthesis of Naphthopyrans
Source: Molecules. 2023 Jul 19;28(14):5502. doi: 10.3390/molecules28145502 (PMC10385152; doi:10.3390/molecules28145502)
Supplement: Supplementary file 1 [file molecules-28-05502-s001.zip › Supporting Information.pdf]

Supporting Information

K_2CO_3-Promoted oxy-Michael Addition/Cyclization of α,β-Unsaturated Carbonyl Compounds with Naphthols: Synthesis of Naphthopyrans

Shan-Shan Li,[†] Li-Li Zhao,[†] Min Pan,[†] Na Feng,[†] Jin-Bao Peng,[†] and Ai-Jun Ma*[†]

[†] School of Biotechnology and Health Sciences, Wuyi University, Jiangmen, Guangdong 529020, People's Republic of China.

E-mail: maaijun@wyu.edu.cn

Content

1. Optimization of Reaction Conditions 3

2. Structure Assignment of X-Ray Crystallographic Analysis 4

3. Copies of NMR Spectra for Compounds 5

4. Copies of HRMS Spectra for Compounds 26

1. Optimization of Reaction Conditions

Table S1. Optimization of Protecting groups.

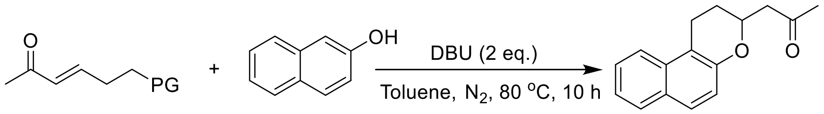

Entry	Protecting groups	Yield (%)[b]
1	OTBDPS	21
2	OBn	trace
3	OAc	30
4	OTBS	31

[a]Standard conditions: naphthalen-2-ol (1.5 equiv), DBU (2 equiv), Toluene (0.5 mL), 80 °C, 10 h. [b]GC yield (*N*-dodecane as the internal standard).

Table S2. Optimization of Solvents.

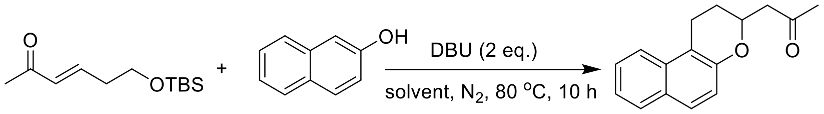

Entry	Solvent	Yield (%)[b]
1	DMSO	17
2	THF	26
3	MeOH	37
4	DMF	41
5	1,4-Dioxane	32
6	Toluene	27
7	DCE	28
8	EA	20

[a]Standard conditions: naphthalen-2-ol (1.5 equiv), DBU (2 equiv), solvent (0.5 mL), 80 °C, 10 h. [b]GC yield (*N*-dodecane as the internal standard).

Table S3. Optimization of Bases.[a]

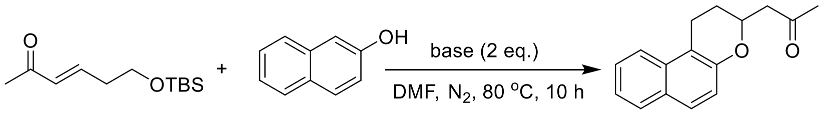

Entry	Base	Yield (%)[b]
1	K_2CO_3	91(88)[c]
2	DBU	39
3	CsF	17
4	K_3PO_4	9
5	*t*-BuOK	0
6	$CsCO_3$	3

[a]Standard conditions: naphthalen-2-ol (1.5 equiv), base (2 equiv), DMF (0.5 mL), 80 °C, 10 h. [b]GC yield (*N*-dodecane as the internal standard).

2. Structure Assignment of X-Ray Crystallographic Analysis

The single crystal of **4ag** which was used for the determination of its relative configurations via X-ray crystallography (see below). The intensity data were collected using graphite-monochromated Mo Kα radiation.

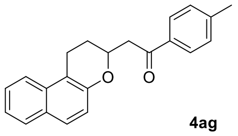

4ag

≡

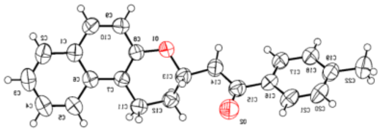

X-ray of **4ag**
CCDC:2171562

Crystal data and structure refinement for 4ag	
Identification code	**4ag**
Empirical formula	$C_{22}H_{20}O_2$
Formula weight	316.38
Temperature/K	292.6(2)
Crystal system	monoclinic
Space group	$P2_1/c$
a/Å	12.7178(2)
b/Å	7.80687(12)
c/Å	17.5718(3)
α/°	90
β/°	103.8395(16)
γ/°	90
Volume/Å^3	1693.99(5)
Z	4
ρ_{calc}g/cm^3	1.241
μ/mm^{-1}	0.614
F(000)	672.0
Crystal size/mm^3	0.14 × 0.13 × 0.12
Radiation	Cu Kα (λ = 1.54184)
2Θ range for data collection/°	7.158 to 151.096
Index ranges	-14 ≤ h ≤ 15, -6 ≤ k ≤ 9, -22 ≤ l ≤ 20
Reflections collected	11569
Independent reflections	3363 [R_{int} = 0.0193, R_{sigma} = 0.0146]
Data/restraints/parameters	3363/0/219
Goodness-of-fit on F^2	1.073
Final R indexes [I>=2σ (I)]	R_1 = 0.0421, wR_2 = 0.1273
Final R indexes [all data]	R_1 = 0.0456, wR_2 = 0.1305
Largest diff. peak/hole / e Å^{-3}	0.19/-0.14

Reference

1. Khrizman, A.; Cheng, H.Y.; Moyna, G. Synthesis of sequentially deuterated 1-*n*-butyl-3-methylimidazolium ionic liquids. *J. Labelled. Compd. Rad*. **2011**, *54*, 401-407.
2. Balasubramanyam, P.; Reddy, G.C.; Salvanna, N.; Das, B. Efficient stereoselective total synthesis of (+)-cryptofolione and the first synthesis of (-)-cryptocaryalactone. *Synthesis*.**2011**, *22*, 3706-3710.
3. Lin, L.; Romano, C.; Mazet, C. Palladium-Catalyzed Long-Range Deconjugative Isomerization of Highly Substituted *α,β*-Unsaturated Carbonyl Compounds. *J. Am. Chem. Soc.* **2016**, *138*, 10344-10350.
4. Wang, L.; Yang, D.; Li, D.; Liu, X.; Wang, P.; Wang, K.; Zhu, H.; Bai, L.; Wang, R. The Important Role of the Byproduct Triphenylphosphine Oxide in the Magnesium(II)-Catalyzed Enantioselective Reaction of Hemiacetals and Phosphorus Ylides. *Angew. Chem., Int. Ed.* **2018**, *57*, 9088-9092.

3. Copies of NMR Spectra for Compounds

3.1. NMR Spectra of **4aa**-**4ak**

The gram-scale reaction: (*E*)-6-((tert-butyldimethylsilyl)oxy)hex-3-en-2-one (0.2 mmol), naphthalen-2-ol (0.3 mmol) and potassium carbonate (0.4 mmol) were added into an round bottom flask which was carried out under N_2 atmosphere. *N,N*-Dimethylformamide were added to the reaction tube. After a time period of 10 h, the solution was diluted with ethyl acetate , washed with brine and concentrated in vacuo. The crude product was purified by column chromatography on silica gel to afford the corresponding product.

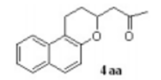

4aa

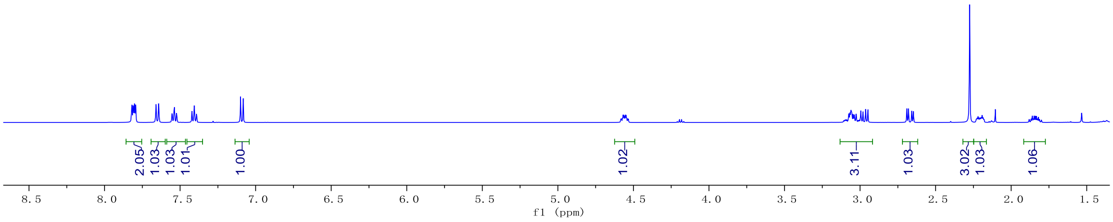

2.05
1.03
1.03
1.01
1.00
1.02
3.11
1.03
3.02
1.03
1.06
8.5 8.0 7.5 7.0 6.5 6.0 5.5 5.0 4.5 4.0 3.5 3.0 2.5 2.0 1.5
f1 (ppm)

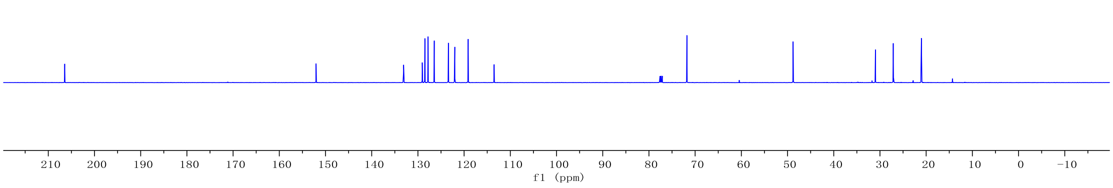

206.4
152.0
133.1
129.1
128.5
127.8
126.5
123.4
122.0
119.1
113.5
71.8
48.8
31.0
27.1
21.0
210 200 190 180 170 160 150 140 130 120 110 100 90 80 70 60 50 40 30 20 10 0 -10
f1 (ppm)

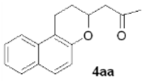

4aa

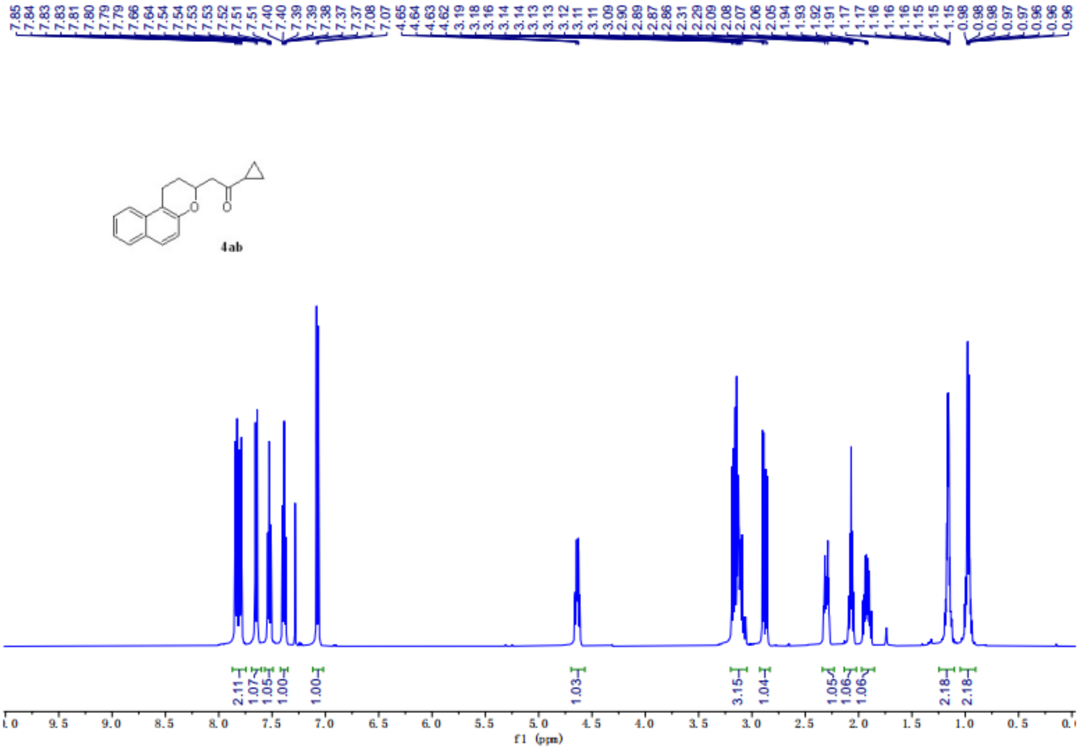
7.85 7.84 7.83 7.81 7.80 7.79 7.79 7.66 7.64 7.54 7.54 7.53 7.52 7.51 7.51 7.40 7.39 7.39 7.38 7.37 7.37 7.08 7.07 4.65 4.64 4.63 4.62 3.19 3.18 3.16 3.14 3.14 3.13 3.13 3.12 3.11 3.11 3.09 2.90 2.89 2.87 2.86 2.31 2.29 2.09 2.08 2.07 2.06 2.05 1.94 1.93 1.92 1.91 1.17 1.17 1.16 1.16 1.15 1.15 1.15 0.98 0.98 0.98 0.97 0.96 0.96 0.96
4ab
2.11 1.07 1.05 1.00 1.00 1.03 3.15 1.04 1.05 1.06 1.06 2.18 2.18
f1 (ppm)

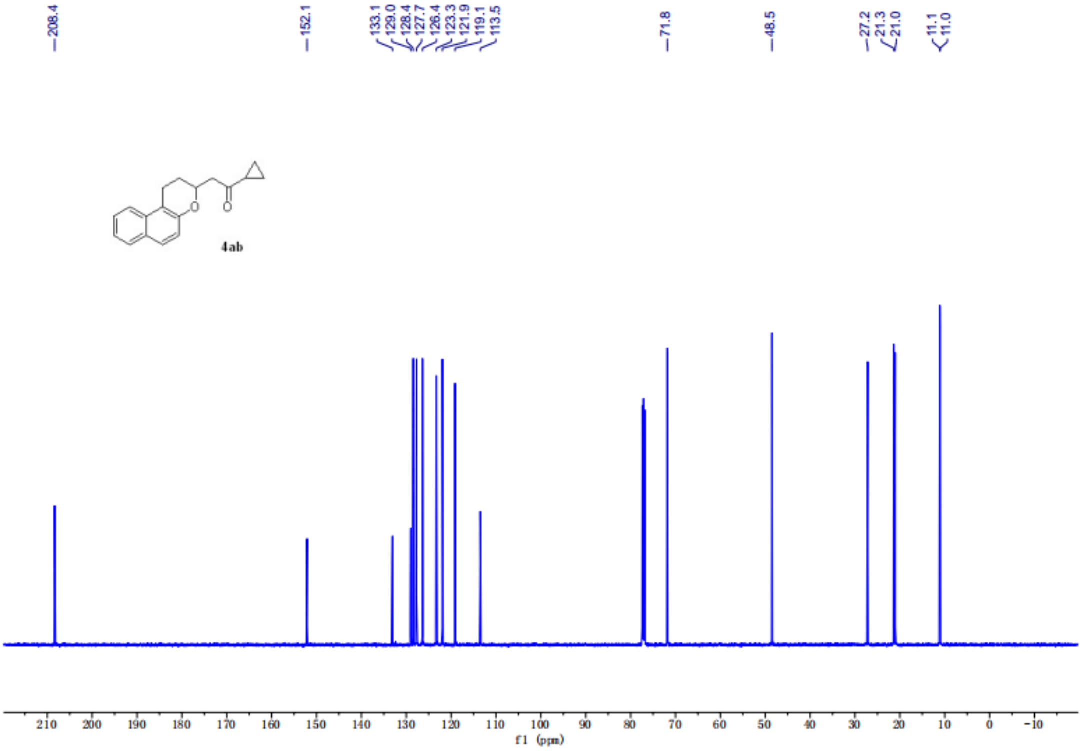
208.4 152.1 133.1 129.0 128.4 127.7 126.4 123.3 121.9 119.1 113.5 71.8 48.5 27.2 21.3 21.0 11.1 11.0
4ab
f1 (ppm)

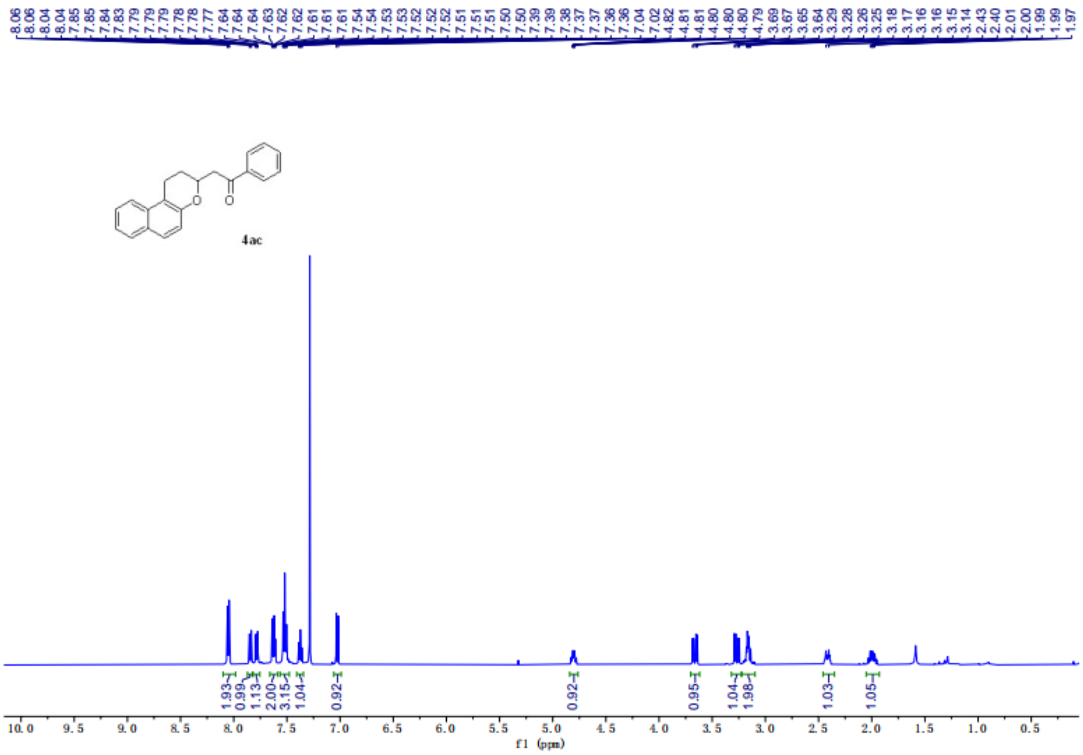
8.06 8.06 8.04 8.04 7.85 7.84 7.83 7.79 7.79 7.79 7.78 7.78 7.77 7.64 7.64 7.64 7.63 7.62 7.62 7.61 7.61 7.61 7.54 7.54 7.53 7.53 7.52 7.52 7.52 7.51 7.51 7.51 7.50 7.50 7.39 7.39 7.38 7.37 7.37 7.36 7.36 7.04 7.02 4.82 4.81 4.81 4.80 4.80 4.79 3.69 3.67 3.65 3.64 3.29 3.28 3.26 3.25 3.18 3.17 3.16 3.15 3.14 2.43 2.40 2.01 2.00 1.99 1.97
4ac
1.93 0.99 1.13 2.00 3.15 1.04 0.92 0.92 0.95 1.04 1.98 1.03 1.05
f1 (ppm)

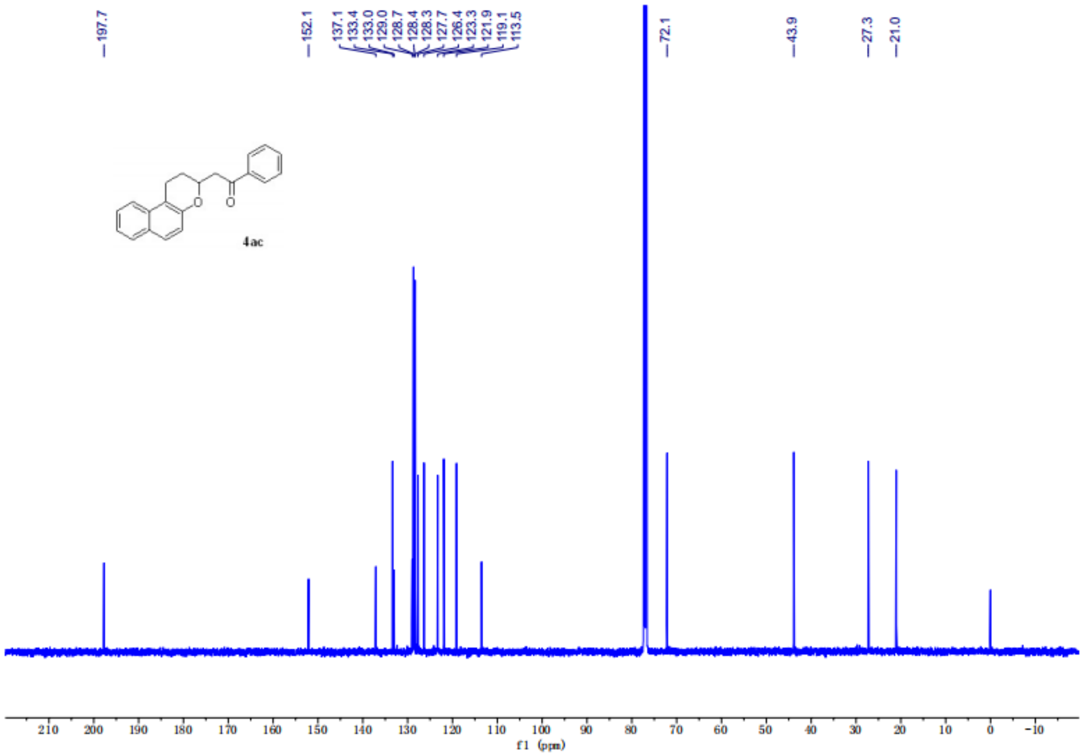
197.7
152.1
137.1 133.4 133.0 129.0 128.7 128.4 128.3 127.7 126.4 123.3 121.9 119.1 113.5
72.1
43.9
27.3
21.0
4ac
f1 (ppm)

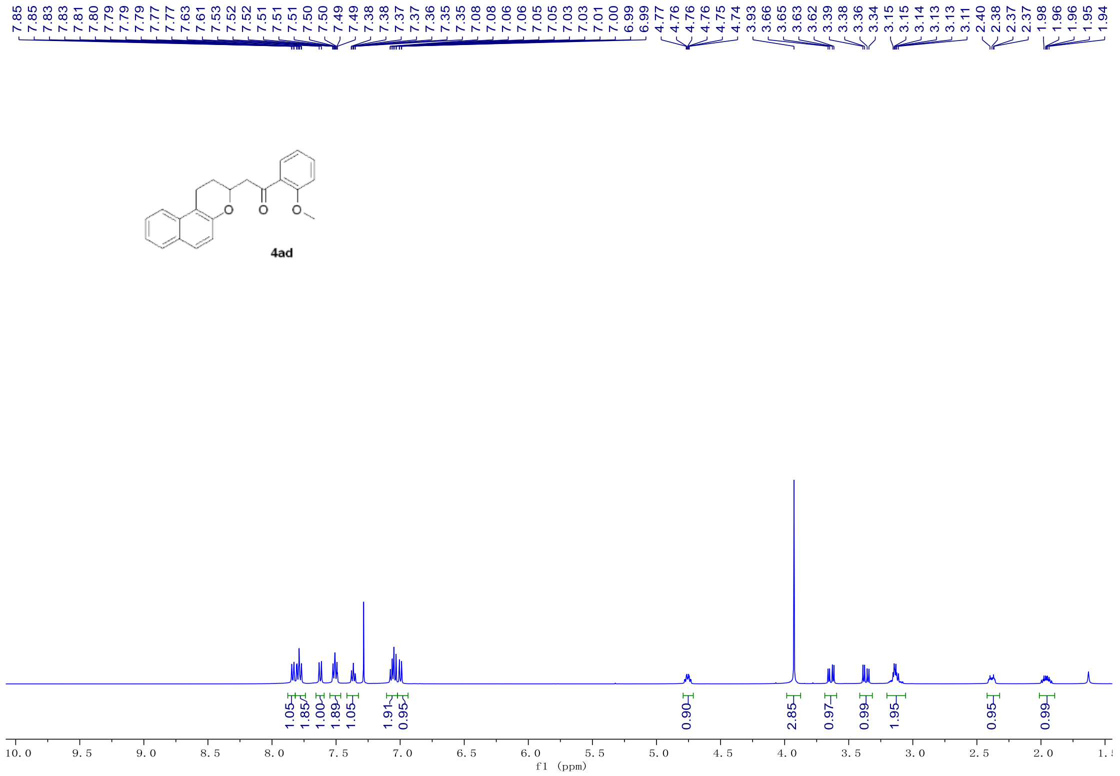
7.85 7.85 7.83 7.83 7.81 7.80 7.79 7.79 7.79 7.77 7.77 7.63 7.61 7.53 7.52 7.52 7.51 7.51 7.51 7.50 7.49 7.49 7.38 7.38 7.37 7.37 7.36 7.35 7.35 7.08 7.08 7.06 7.06 7.05 7.05 7.03 7.03 7.01 7.00 6.99 6.99 4.77 4.76 4.76 4.76 4.75 4.74 3.93 3.66 3.65 3.63 3.62 3.39 3.38 3.36 3.34 3.15 3.15 3.14 3.13 3.13 3.11 2.40 2.38 2.37 2.37 1.98 1.96 1.96 1.95 1.94
4ad
1.05 1.85 1.00 1.89 1.05 1.91 0.95 0.90 2.85 0.97 0.99 1.95 0.95 0.99
10.0 9.5 9.0 8.5 8.0 7.5 7.0 6.5 6.0 5.5 5.0 4.5 4.0 3.5 3.0 2.5 2.0
f1 (ppm)

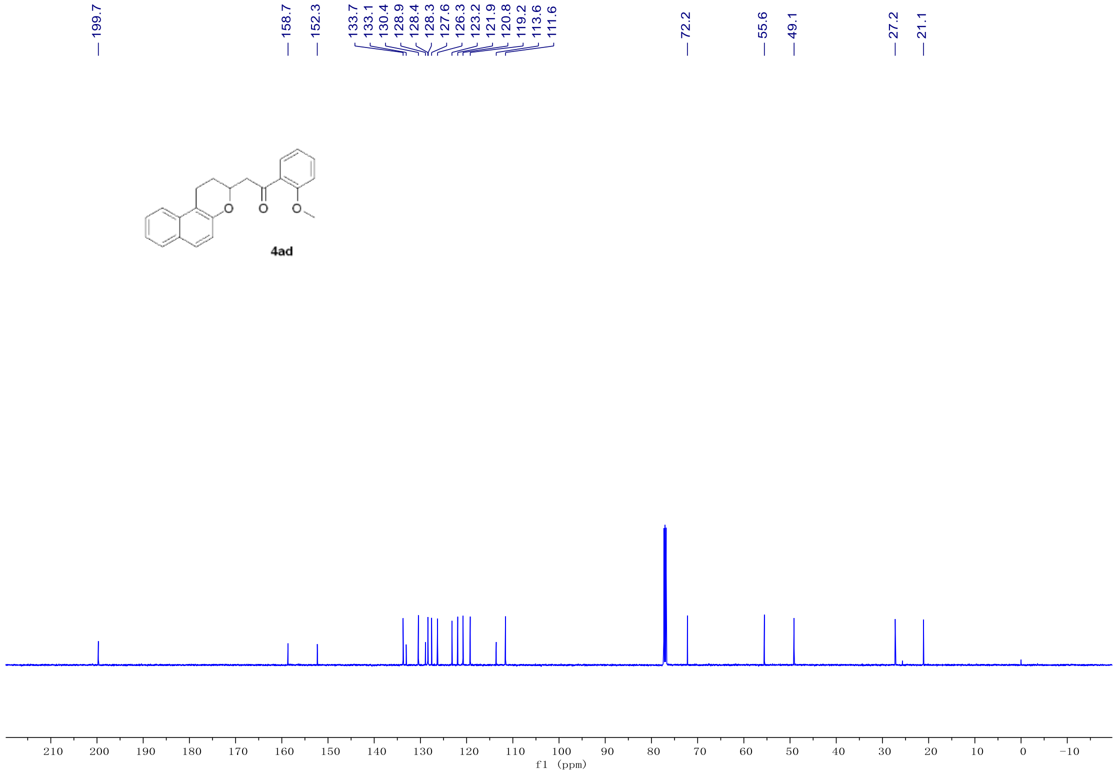
199.7 158.7 152.3 133.7 133.1 130.4 128.9 128.4 128.3 127.6 126.3 123.2 121.9 120.8 119.2 113.6 111.6 72.2 55.6 49.1 27.2 21.1
4ad
210 200 190 180 170 160 150 140 130 120 110 100 90 80 70 60 50 40 30 20 10 0 −10
f1 (ppm)

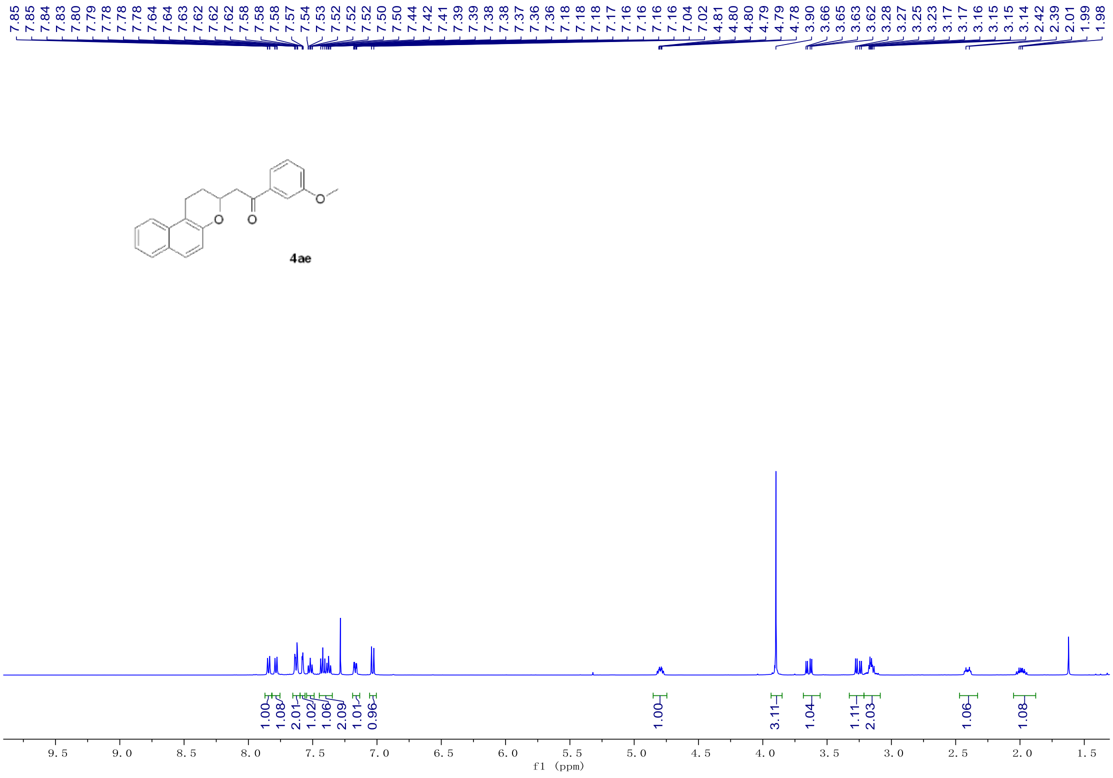

7.85 7.85 7.84 7.83 7.80 7.79 7.78 7.78 7.78 7.64 7.63 7.62 7.62 7.62 7.58 7.58 7.58 7.57 7.54 7.53 7.52 7.52 7.50 7.44 7.42 7.41 7.39 7.39 7.38 7.38 7.37 7.36 7.36 7.18 7.18 7.17 7.16 7.16 7.16 7.16 7.04 7.02 4.81 4.80 4.80 4.79 4.78 3.90 3.66 3.65 3.63 3.62 3.28 3.27 3.25 3.23 3.17 3.17 3.16 3.15 3.15 3.14 2.42 2.39 2.01 1.99 1.98
O
4ae
1.00 1.08 2.01 1.02 1.06 2.09 1.01 0.96 1.00 3.11 1.04 1.11 2.03 1.06 1.08
9.5 9.0 8.5 8.0 7.5 7.0 6.5 6.0 5.5 5.0 4.5 4.0 3.5 3.0 2.5 2.0 1.5
f1 (ppm)

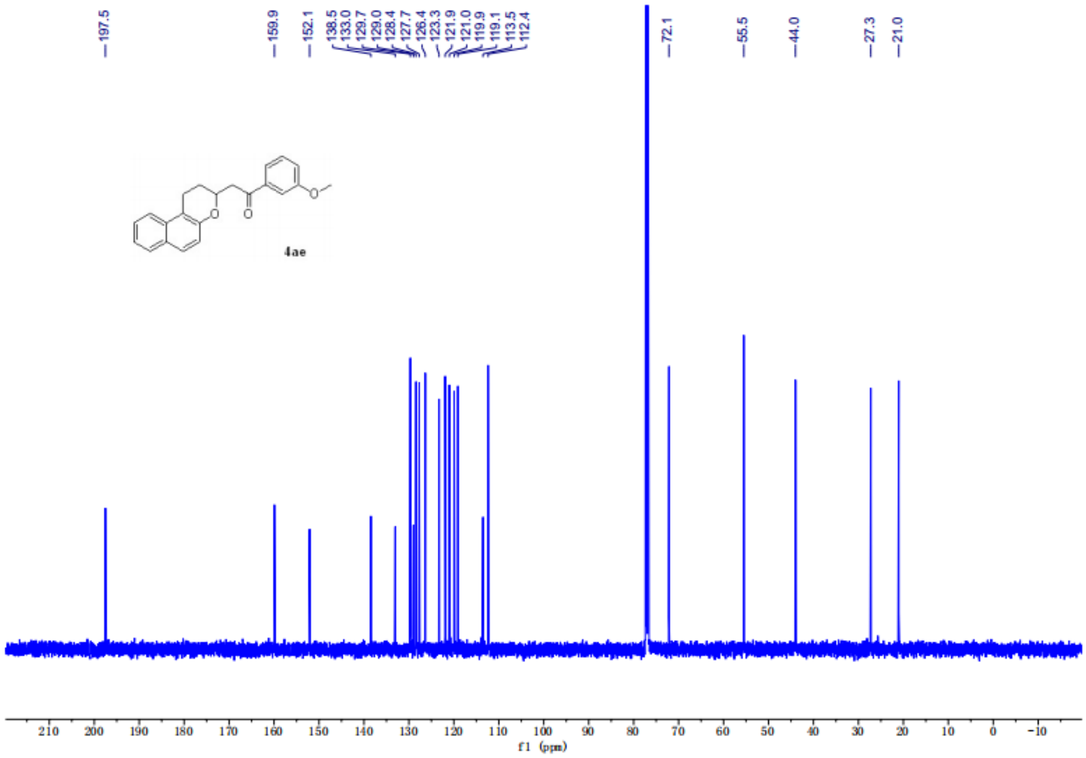

197.5 159.9 152.1 138.5 133.0 129.7 129.0 128.4 127.7 126.4 123.3 121.9 121.0 119.9 119.1 113.5 112.4 72.1 55.5 44.0 27.3 21.0
O
4ae
210 200 190 180 170 160 150 140 130 120 110 100 90 80 70 60 50 40 30 20 10 0 -10
f1 (ppm)

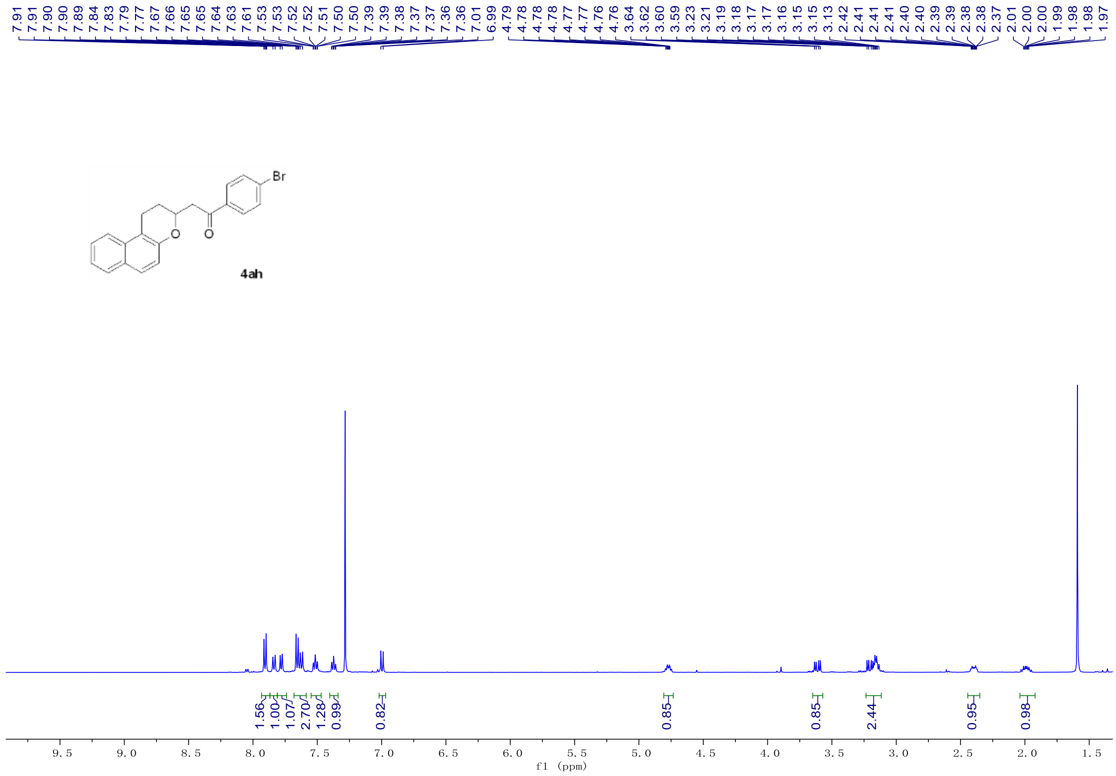
7.91 7.91 7.90 7.90 7.89 7.84 7.83 7.79 7.77 7.67 7.66 7.65 7.65 7.64 7.63 7.61 7.53 7.53 7.52 7.52 7.51 7.50 7.50 7.39 7.39 7.38 7.37 7.37 7.36 7.36 7.01 6.99 4.79 4.78 4.78 4.77 4.77 4.76 4.76 3.64 3.62 3.60 3.59 3.23 3.21 3.19 3.18 3.17 3.16 3.15 3.15 3.13 2.42 2.41 2.41 2.41 2.40 2.39 2.39 2.38 2.38 2.37 2.01 2.00 2.00 1.99 1.98 1.98 1.97
Br
O
O
4ah
1.56 1.00 1.07 2.70 1.28 0.99 0.82 0.85 0.85 2.44 0.95 0.98
9.5 9.0 8.5 8.0 7.5 7.0 6.5 6.0 5.5 5.0 4.5 4.0 3.5 3.0 2.5 2.0 1.5
f1 (ppm)

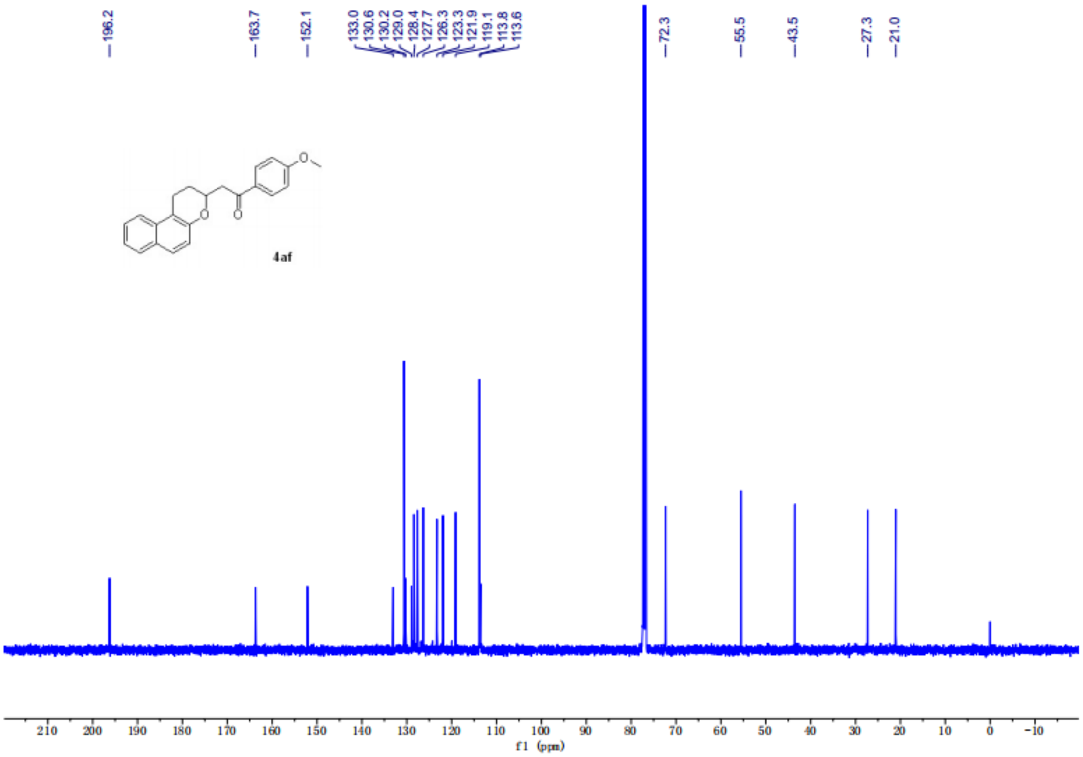
196.2 163.7 152.1 133.0 130.6 130.2 129.0 128.4 127.7 126.3 123.3 121.9 119.1 113.8 113.6 72.3 55.5 43.5 27.3 21.0
O
O
O
4af
210 200 190 180 170 160 150 140 130 120 110 100 90 80 70 60 50 40 30 20 10 0 -10
f1 (ppm)

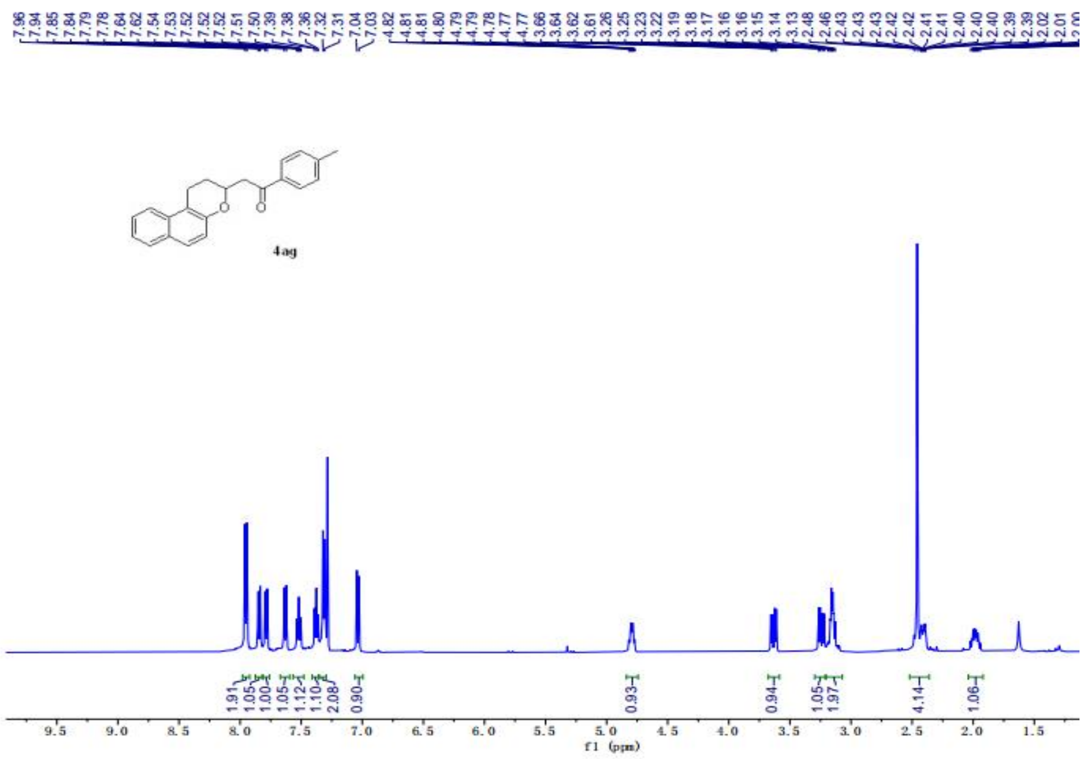
7.96 7.94 7.85 7.84 7.79 7.78 7.64 7.62 7.54 7.53 7.52 7.52 7.51 7.50 7.39 7.38 7.36 7.32 7.31 7.04 7.03 4.82 4.81 4.80 4.79 4.79 4.78 4.77 4.77 3.66 3.64 3.62 3.61 3.26 3.25 3.23 3.22 3.19 3.18 3.17 3.16 3.16 3.15 3.14 3.13 2.48 2.46 2.43 2.43 2.42 2.42 2.41 2.41 2.40 2.40 2.39 2.02 2.01
4ag
1.91 1.05 1.00 1.05 1.12 1.10 2.08 0.90 0.93 0.94 1.05 1.97 4.14 1.06
9.5 9.0 8.5 8.0 7.5 7.0 6.5 6.0 5.5 5.0 4.5 4.0 3.5 3.0 2.5 2.0 1.5
f1 (ppm)

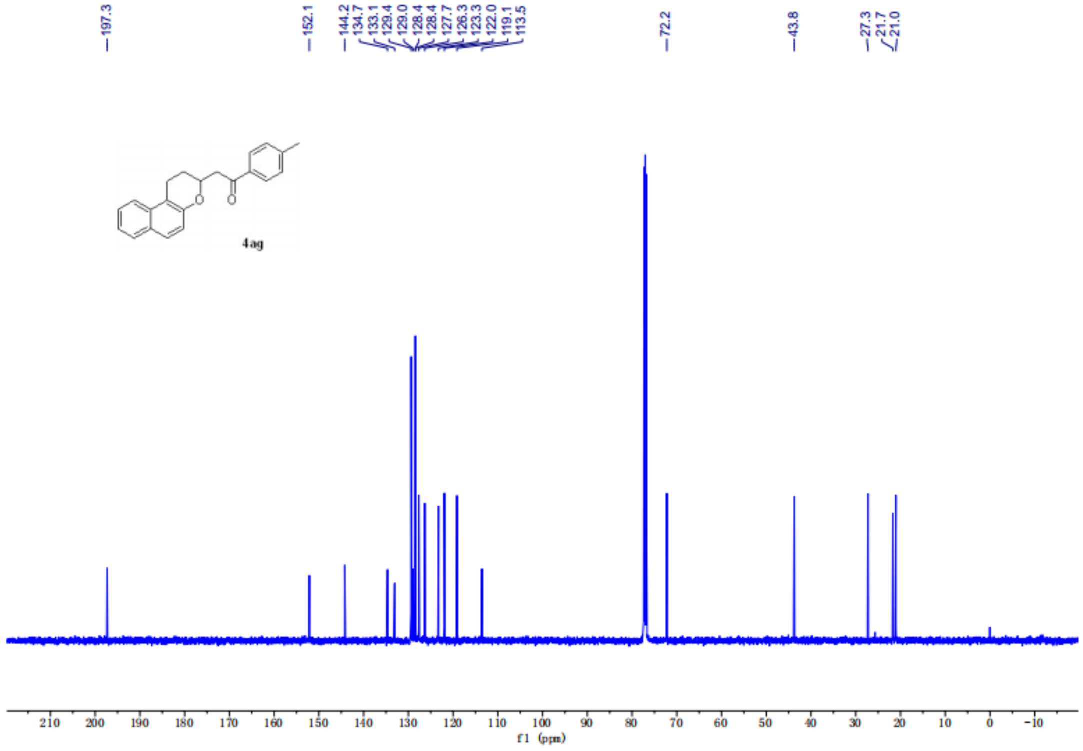
197.3 152.1 144.2 134.7 133.1 129.4 129.0 128.4 128.4 127.7 126.3 123.3 122.0 119.1 113.5 72.2 43.8 27.3 21.7 21.0
4ag
210 200 190 180 170 160 150 140 130 120 110 100 90 80 70 60 50 40 30 20 10 0 -10
f1 (ppm)

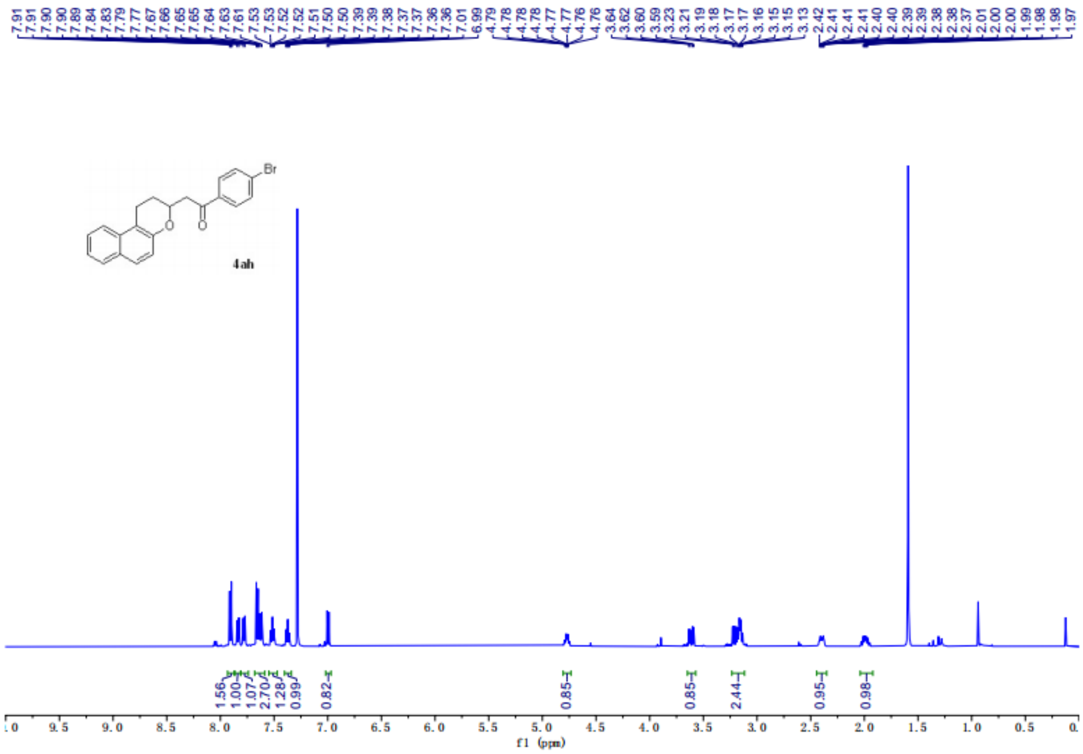
4ah
f1 (ppm)

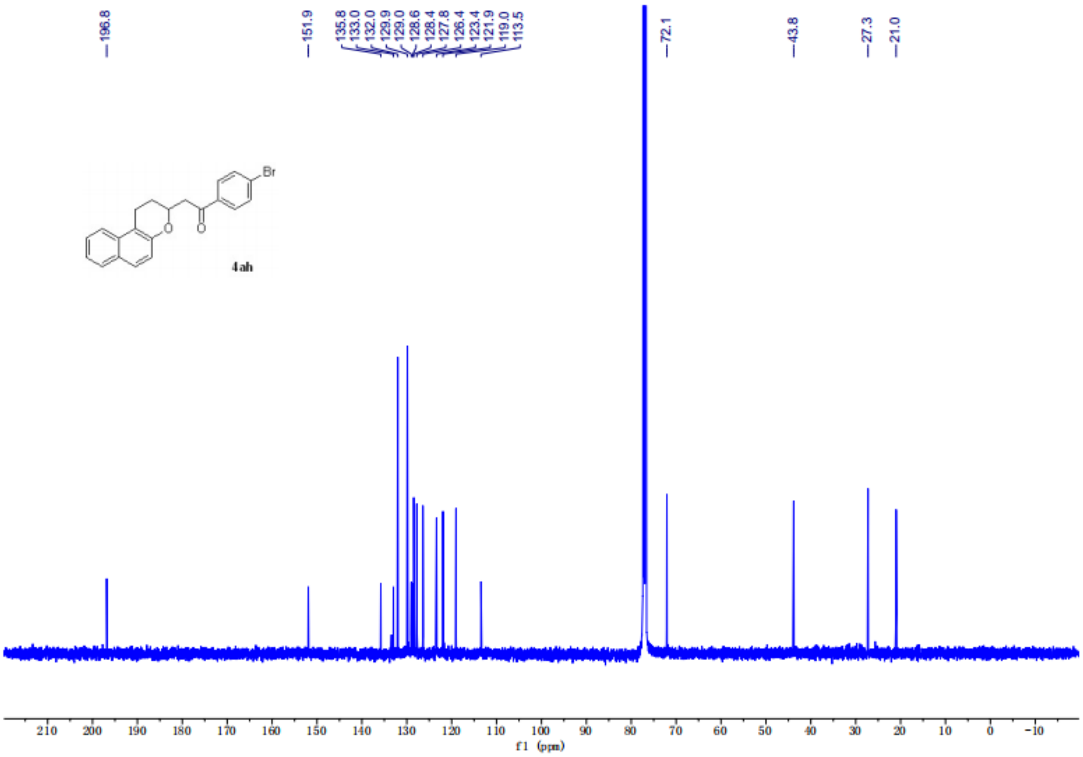
4ah
f1 (ppm)

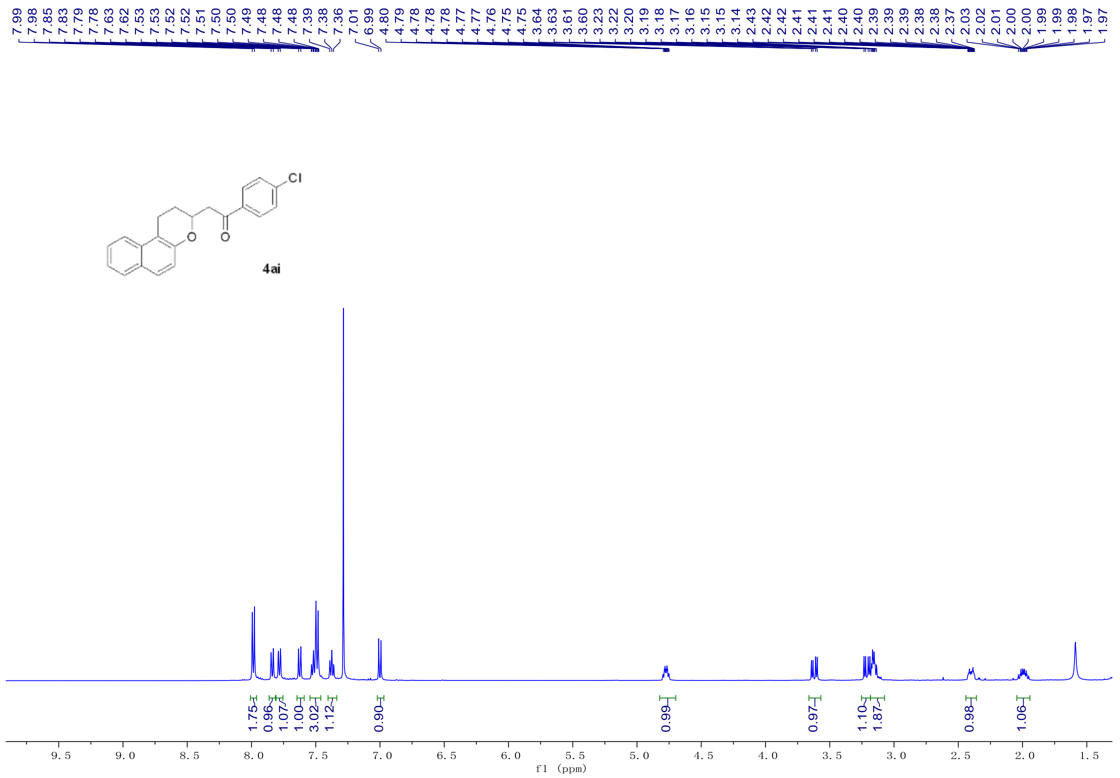
7.99 7.98 7.85 7.83 7.79 7.78 7.63 7.62 7.53 7.53 7.52 7.52 7.51 7.50 7.50 7.49 7.48 7.48 7.48 7.39 7.38 7.36 7.01 6.99 4.80 4.79 4.78 4.78 4.77 4.77 4.76 4.75 4.75 3.64 3.63 3.61 3.60 3.23 3.22 3.20 3.19 3.18 3.17 3.16 3.15 3.15 3.14 2.43 2.42 2.42 2.41 2.41 2.40 2.39 2.39 2.39 2.38 2.38 2.37 2.03 2.02 2.01 2.00 2.00 1.99 1.99 1.98 1.97 1.97
Cl
O
O
4ai
1.75 0.96 1.07 1.00 3.02 1.12 0.90 0.99 0.97 1.10 1.87 0.98 1.06
9.5 9.0 8.5 8.0 7.5 7.0 6.5 6.0 5.5 5.0 4.5 4.0 3.5 3.0 2.5 2.0 1.5
f1 (ppm)

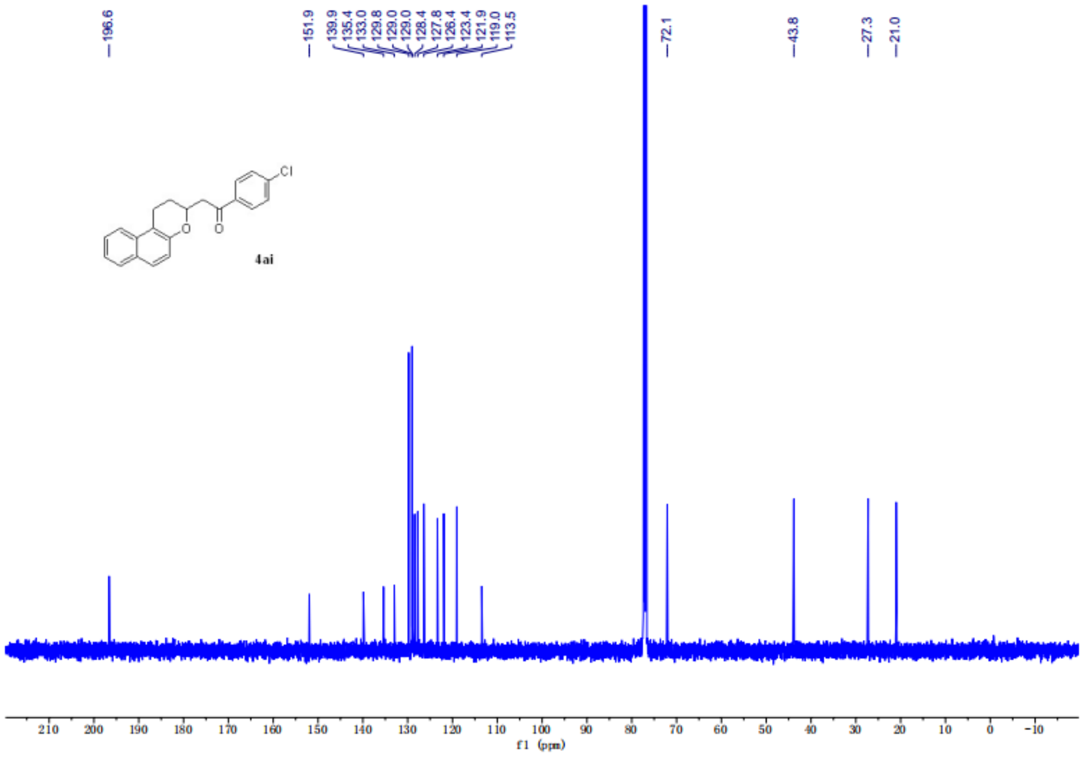
196.6
151.9 139.9 135.4 133.0 129.8 129.0 129.0 128.4 127.8 126.4 123.4 121.9 119.0 113.5
72.1
43.8
27.3
21.0
Cl
O
O
4ai
210 200 190 180 170 160 150 140 130 120 110 100 90 80 70 60 50 40 30 20 10 0 -10
f1 (ppm)

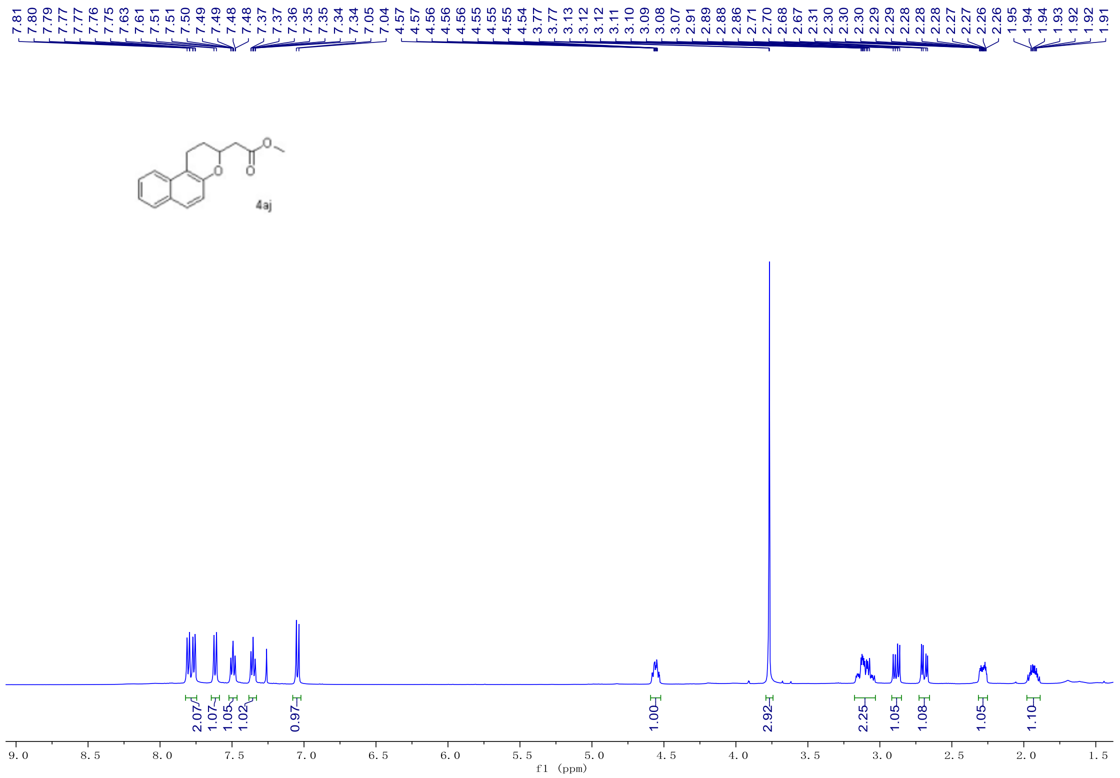
7.81 7.80 7.79 7.77 7.77 7.76 7.75 7.63 7.61 7.51 7.50 7.49 7.49 7.48 7.48 7.37 7.37 7.36 7.35 7.35 7.34 7.34 7.05 7.04 4.57 4.57 4.56 4.56 4.55 4.55 4.55 4.54 3.77 3.77 3.13 3.12 3.12 3.11 3.10 3.09 3.08 3.07 2.91 2.89 2.88 2.86 2.71 2.70 2.68 2.67 2.31 2.30 2.30 2.29 2.29 2.28 2.28 2.27 2.27 2.26 2.26 1.95 1.94 1.94 1.93 1.92 1.92 1.91
O
O
O
4aj
2.07
1.07
1.05
1.02
0.97
1.00
2.92
2.25
1.05
1.08
1.05
1.10
f1 (ppm)

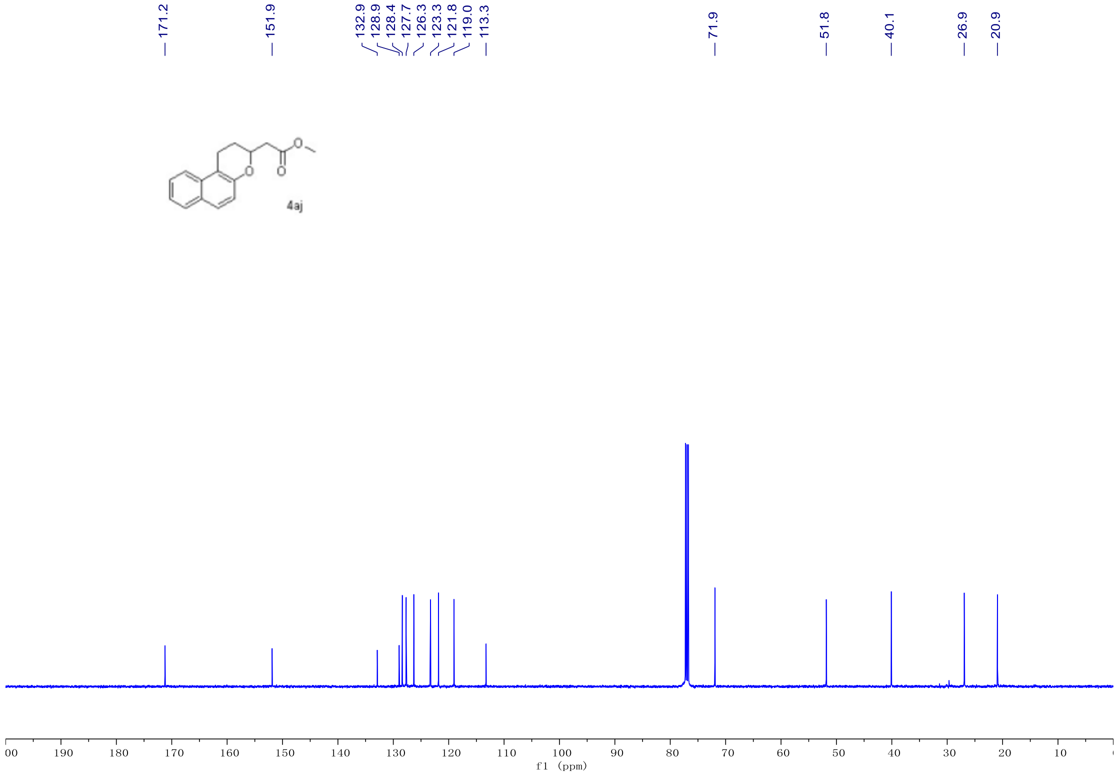
171.2
151.9
132.9 128.9 128.4 127.7 126.3 123.3 121.8 119.0 113.3
71.9
51.8
40.1
26.9
20.9
O
O
O
4aj
f1 (ppm)

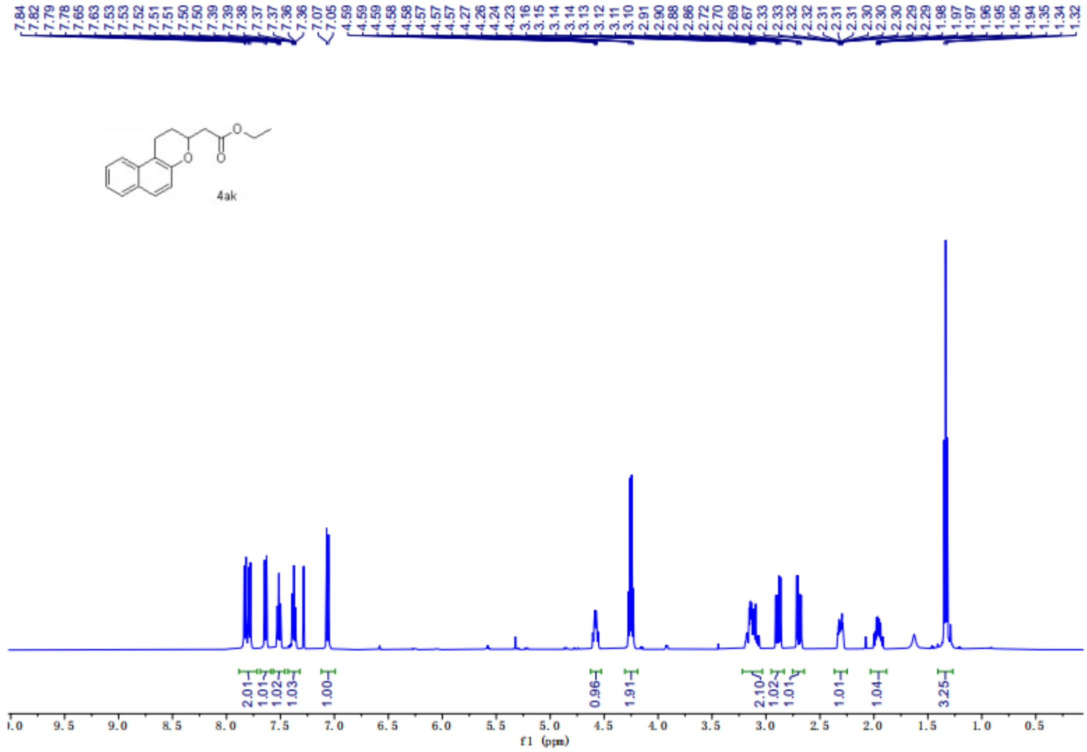
7.84 7.82 7.79 7.78 7.65 7.63 7.53 7.53 7.52 7.51 7.51 7.50 7.50 7.39 7.39 7.38 7.37 7.37 7.36 7.36 7.07 7.05 4.59 4.59 4.58 4.58 4.57 4.57 4.57 4.27 4.26 4.24 4.23 3.16 3.15 3.14 3.14 3.13 3.12 3.11 3.10 2.91 2.90 2.88 2.86 2.72 2.70 2.69 2.67 2.33 2.33 2.32 2.32 2.31 2.31 2.31 2.30 2.30 2.29 2.29 1.98 1.97 1.97 1.96 1.95 1.95 1.94 1.35 1.34 1.32
4ak
2.01 1.01 1.02 1.03 1.00 0.96 1.91 2.10 1.02 1.01 1.01 1.04 3.25
f1 (ppm)

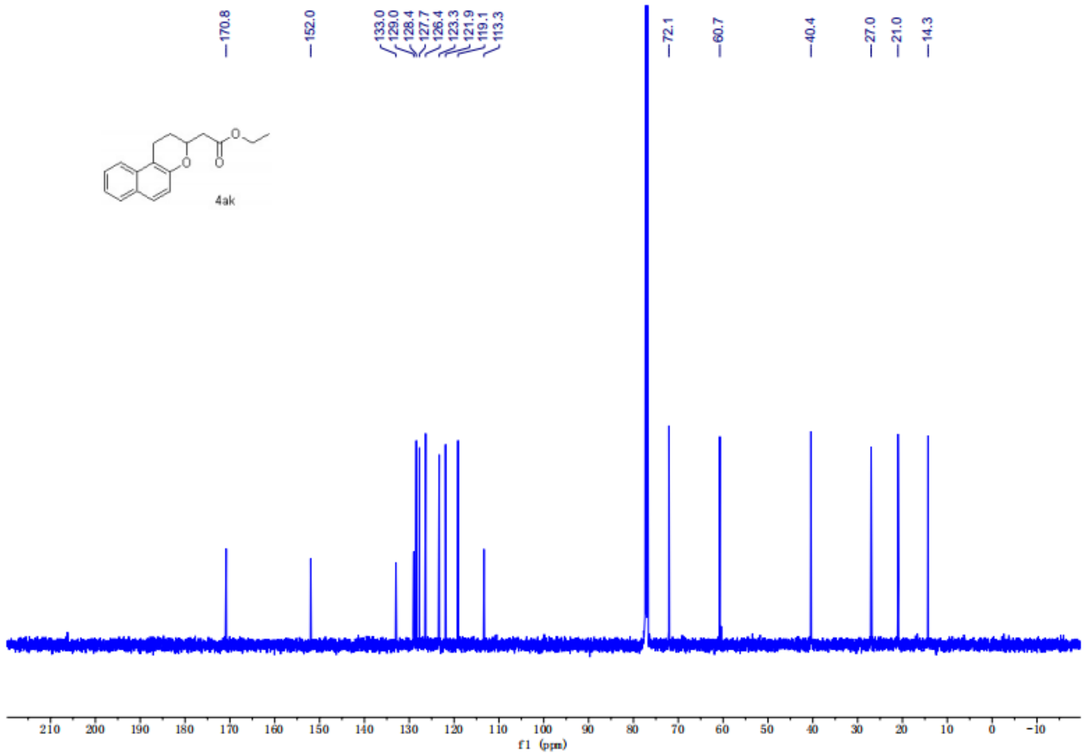
170.8
152.0
133.0 129.0 128.4 127.7 126.4 123.3 121.9 119.1 113.3
72.1
60.7
40.4
27.0
21.0
14.3
4ak
f1 (ppm)

3.2. NMR Spectra of **4ba-4ja**

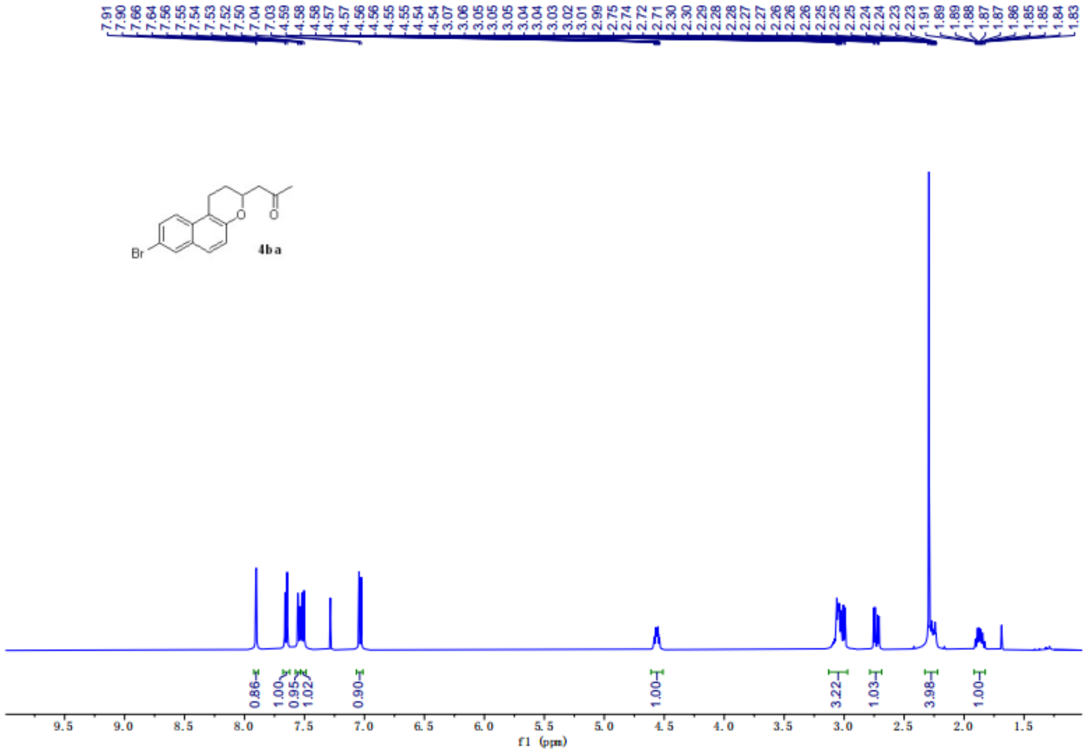

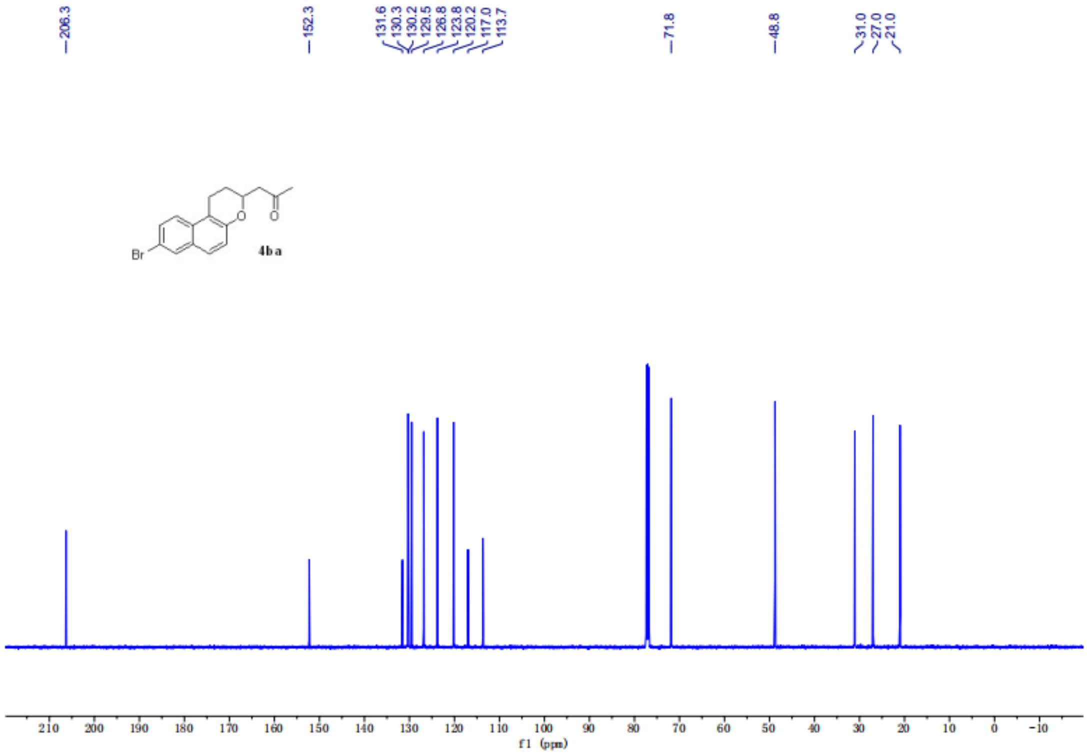

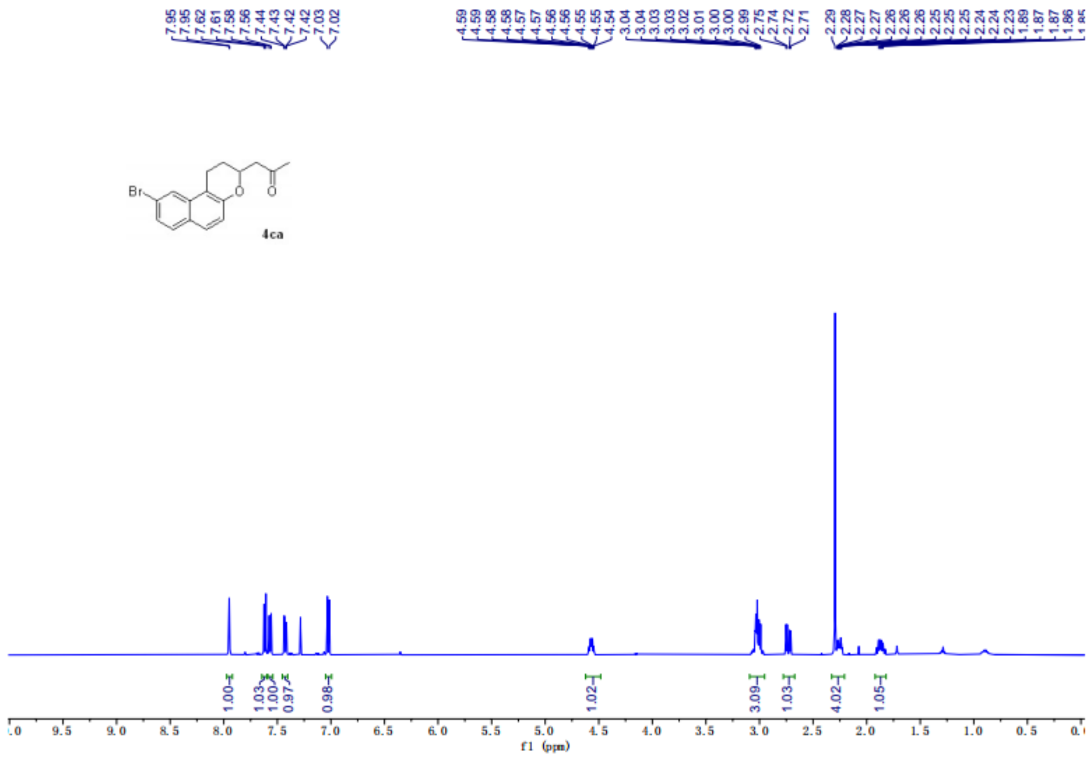
7.95 7.95 7.62 7.61 7.58 7.56 7.44 7.43 7.42 7.42 7.03 7.02
4.59 4.59 4.58 4.58 4.57 4.57 4.56 4.56 4.55 4.55 4.54 3.04 3.04 3.03 3.03 3.02 3.01 3.00 3.00 2.99 2.75 2.74 2.72 2.71
2.29 2.28 2.27 2.27 2.26 2.26 2.26 2.25 2.25 2.25 2.24 2.24 2.23 1.89 1.87 1.87 1.86
Br
O
O
4ca
1.00 1.03 1.00 0.97 0.98 1.02 3.09 1.03 4.02 1.05
f1 (ppm)

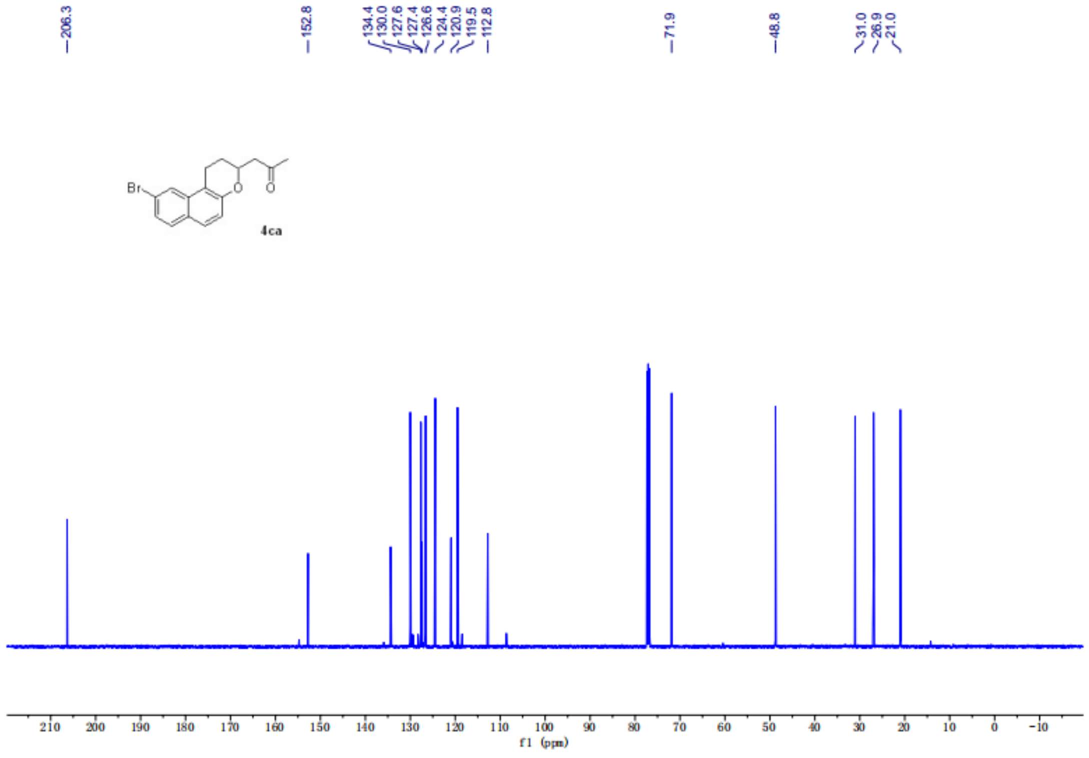
206.3
152.8
134.4 130.0 127.6 127.4 126.6 124.4 120.9 119.5 112.8
71.9
48.8
31.0 26.9 21.0
Br
O
O
4ca
f1 (ppm)

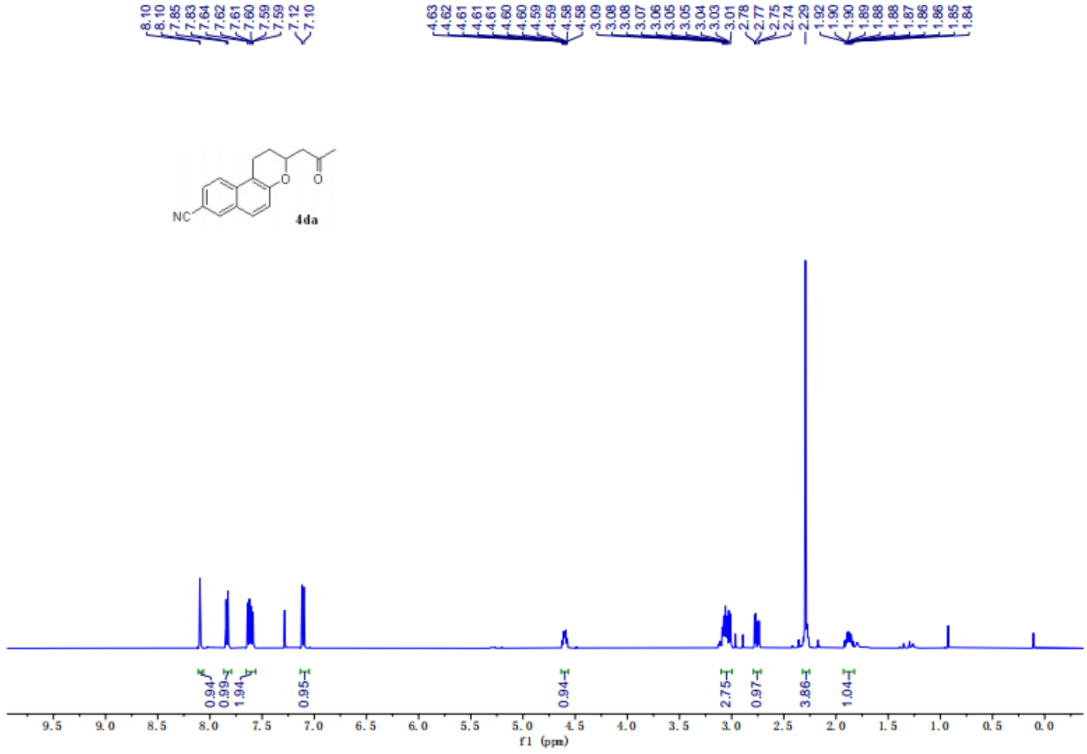
8.10
8.10
7.85
7.83
7.64
7.62
7.61
7.60
7.59
7.59
7.12
7.10
4.63
4.62
4.61
4.61
4.60
4.60
4.59
4.59
4.58
4.58
3.09
3.08
3.08
3.07
3.06
3.05
3.05
3.04
3.03
3.01
2.78
2.77
2.75
2.74
2.29
1.92
1.90
1.90
1.89
1.88
1.88
1.87
1.86
1.85
1.84
NC
O
O
4da
0.94
0.99
1.94
0.95
0.94
2.75
0.97
3.86
1.04
9.5
9.0
8.5
8.0
7.5
7.0
6.5
6.0
5.5
5.0
4.5
4.0
3.5
3.0
2.5
2.0
1.5
1.0
0.5
0.0
f1 (ppm)

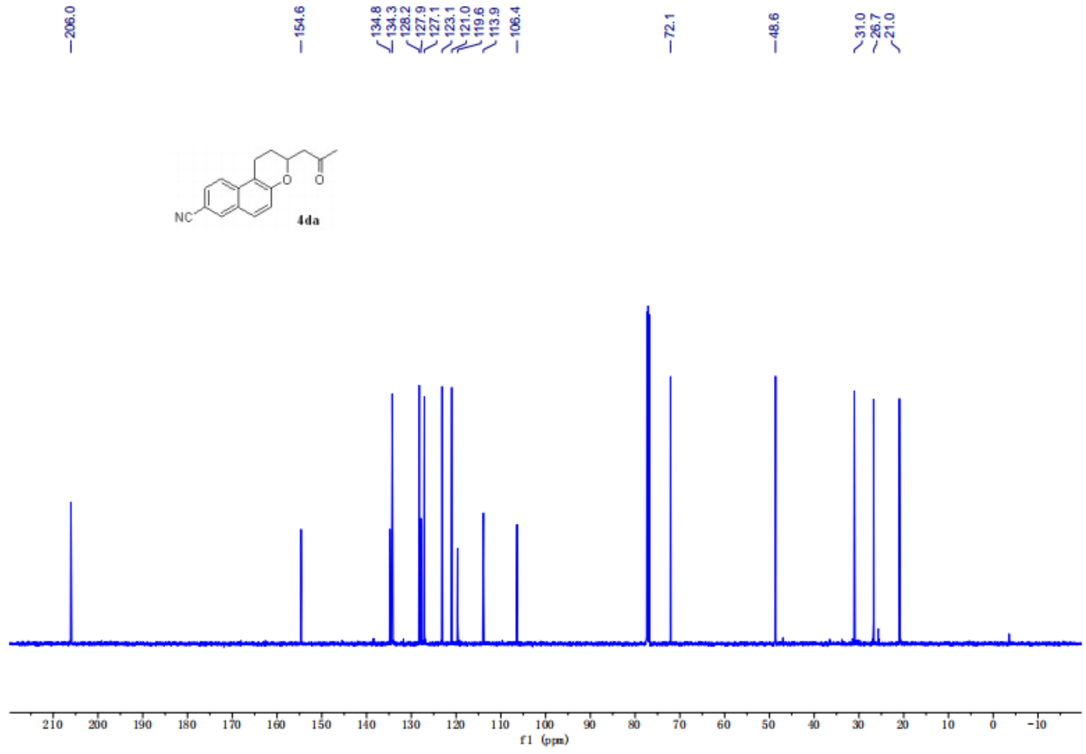
206.0
154.6
134.8
134.3
128.2
127.9
127.1
123.1
121.0
119.6
113.9
106.4
72.1
48.6
31.0
26.7
21.0
NC
O
O
4da
210
200
190
180
170
160
150
140
130
120
110
100
90
80
70
60
50
40
30
20
10
0
-10
f1 (ppm)

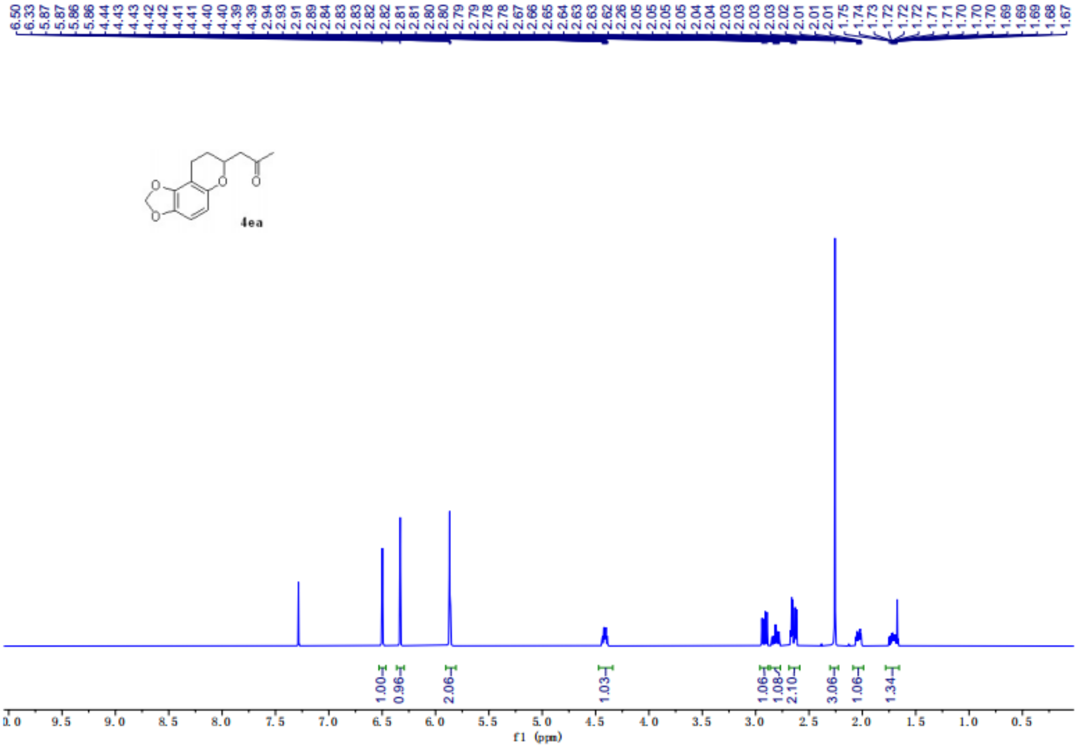
6.50
6.33
5.87
5.86
4.44
4.43
4.43
4.42
4.42
4.41
4.41
4.40
4.40
4.39
4.39
2.94
2.93
2.91
2.89
2.84
2.83
2.83
2.82
2.82
2.81
2.81
2.80
2.80
2.79
2.79
2.78
2.78
2.67
2.66
2.65
2.64
2.63
2.63
2.62
2.26
2.05
2.05
2.05
2.05
2.04
2.04
2.03
2.03
2.03
2.03
2.02
2.01
2.01
1.75
1.74
1.73
1.72
1.72
1.71
1.71
1.70
1.70
1.69
1.69
1.68
1.67
4ea
1.00
0.96
2.06
1.03
1.06
1.08
2.10
3.06
1.06
1.34
f1 (ppm)

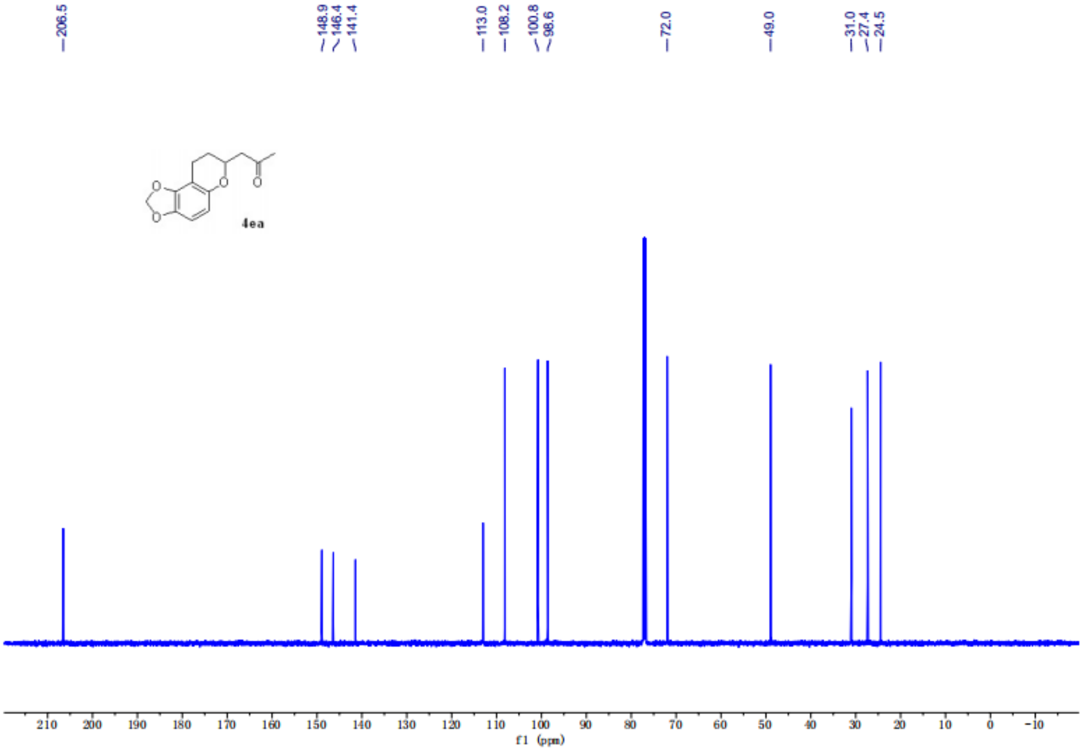
206.5
148.9
146.4
141.4
113.0
108.2
100.8
98.6
72.0
49.0
31.0
27.4
24.5
4ea
f1 (ppm)

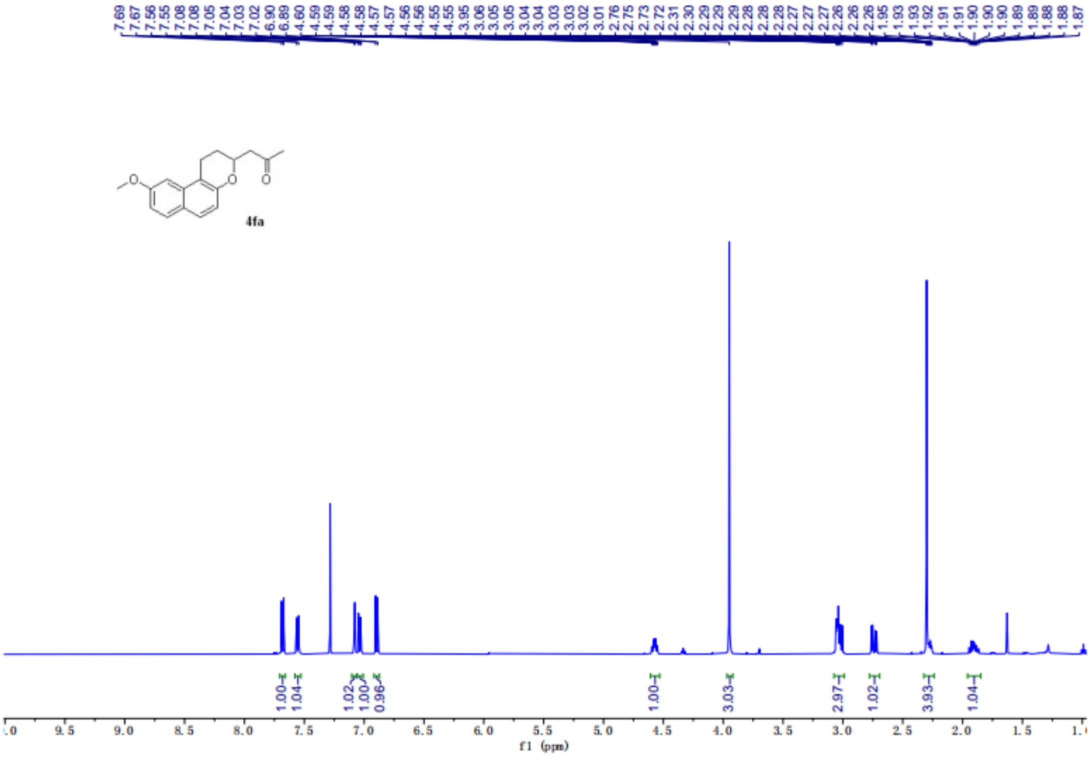
7.69 7.67 7.56 7.55 7.08 7.08 7.05 7.04 7.03 7.02 6.90 6.89 4.60 4.59 4.59 4.58 4.58 4.57 4.57 4.56 4.56 4.55 4.55 3.95 3.06 3.05 3.05 3.04 3.04 3.03 3.03 3.02 3.01 2.76 2.75 2.73 2.72 2.31 2.30 2.29 2.29 2.29 2.28 2.28 2.28 2.27 2.27 2.27 2.26 2.26 2.26 1.95 1.93 1.93 1.92 1.91 1.91 1.90 1.90 1.90 1.89 1.89 1.88 1.87
O
O
O
4fa
1.00 1.04 1.02 1.00 0.96 1.00 3.03 2.97 1.02 3.93 1.04
f1 (ppm)

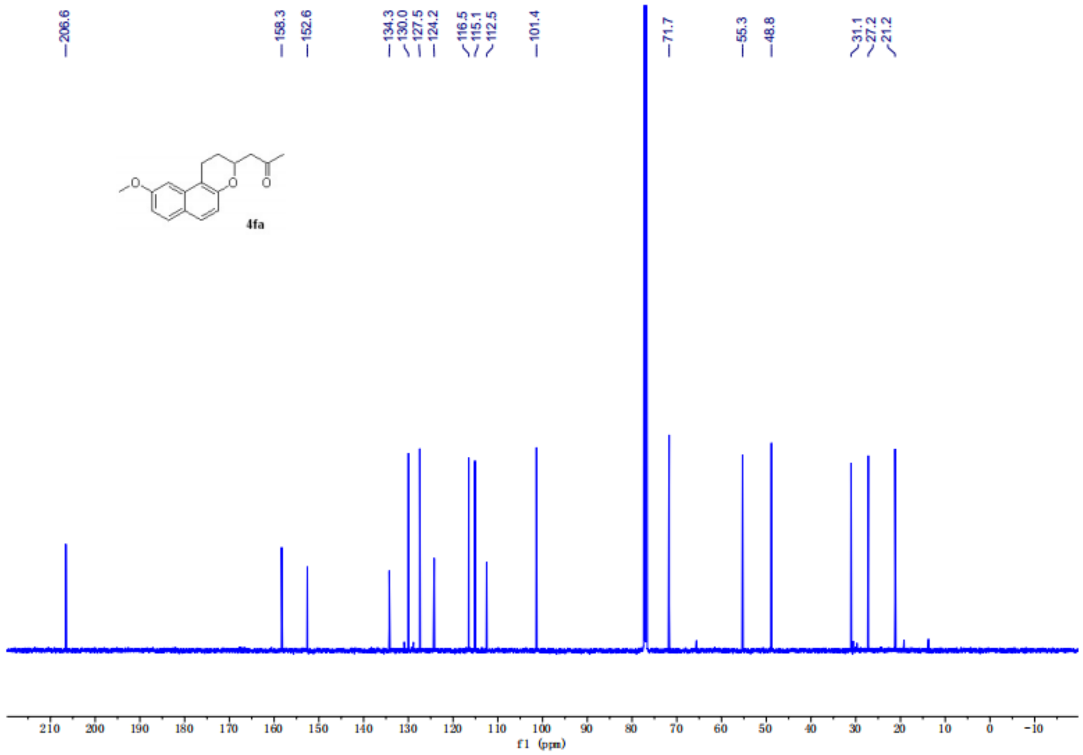
206.6
158.3
152.6
134.3 130.0 127.5 124.2
116.5 115.1 112.5
101.4
71.7
55.3
48.8
31.1 27.2 21.2
O
O
O
4fa
f1 (ppm)

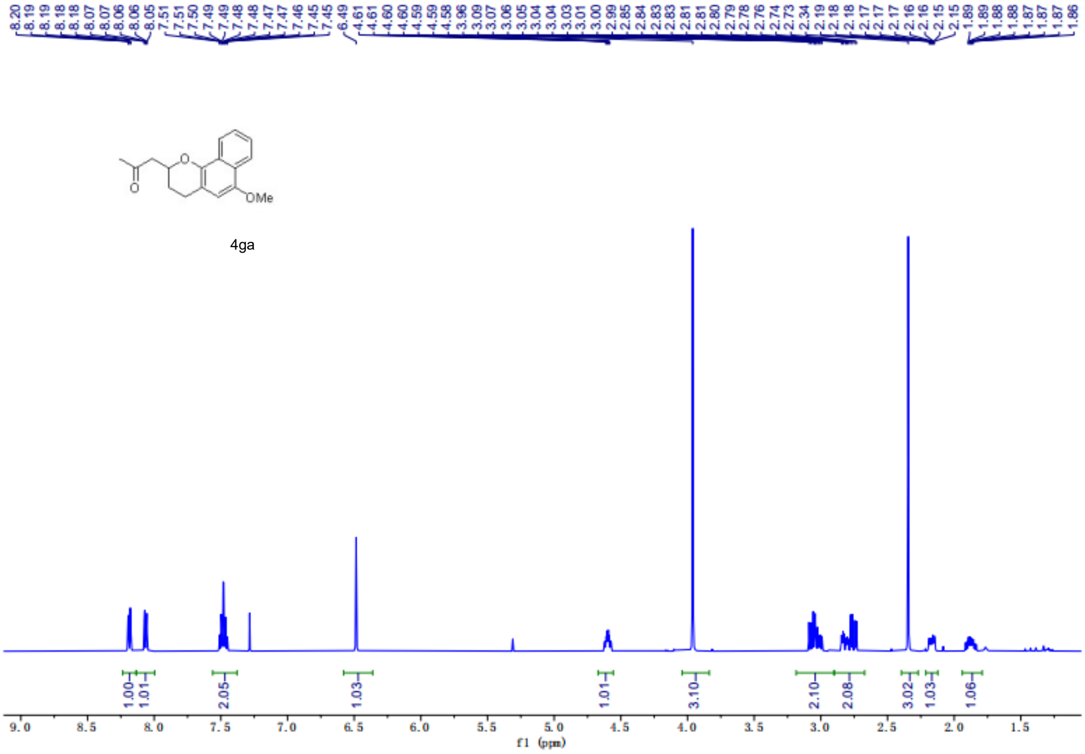
8.20
8.19
8.19
8.18
8.18
8.07
8.07
8.06
8.05
7.51
7.50
7.49
7.49
7.48
7.47
7.46
7.45
7.45
6.49
4.61
4.61
4.60
4.59
4.59
4.58
3.96
3.09
3.07
3.06
3.05
3.04
3.03
3.01
3.00
2.99
2.85
2.84
2.83
2.83
2.81
2.81
2.80
2.79
2.78
2.76
2.74
2.73
2.34
2.19
2.18
2.18
2.17
2.17
2.16
2.16
2.15
1.89
1.88
1.88
1.87
1.87
1.86
1.00
1.01
2.05
1.03
1.01
3.10
2.10
2.08
3.02
1.03
1.06
f1 (ppm)
OMe

4ga

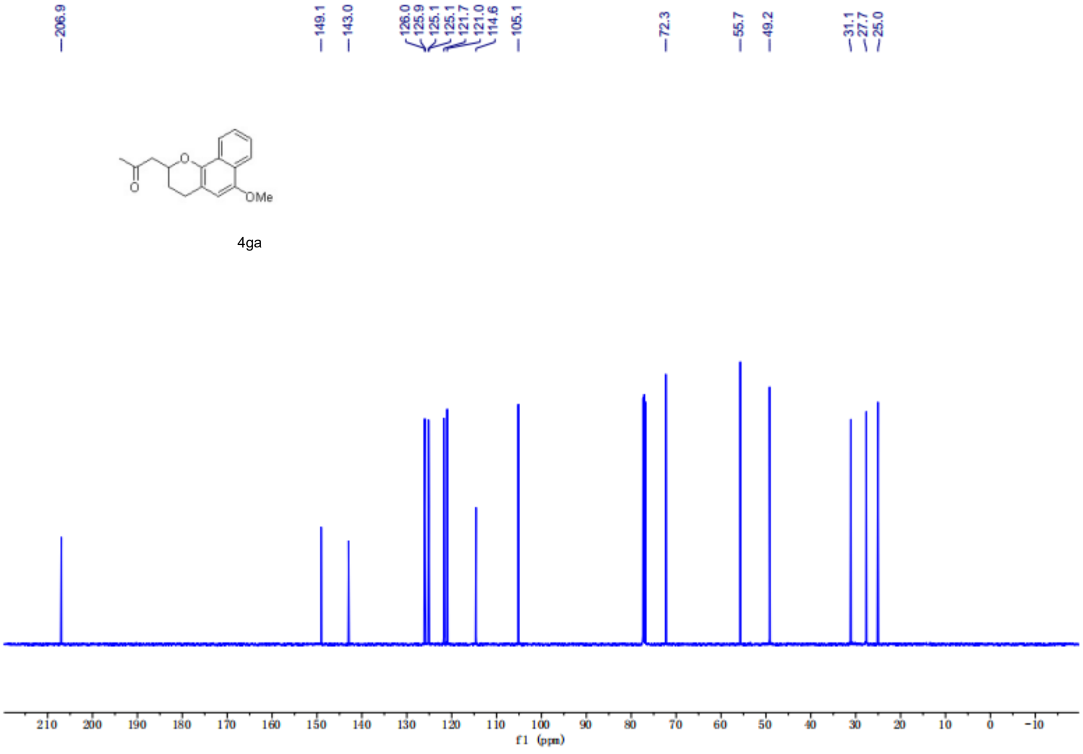
206.9
149.1
143.0
126.0
125.9
125.1
125.1
121.7
121.0
114.6
105.1
72.3
55.7
49.2
31.1
27.7
25.0
f1 (ppm)
OMe

4ga

3.3. NMR Spectra of **5aa-5ab**

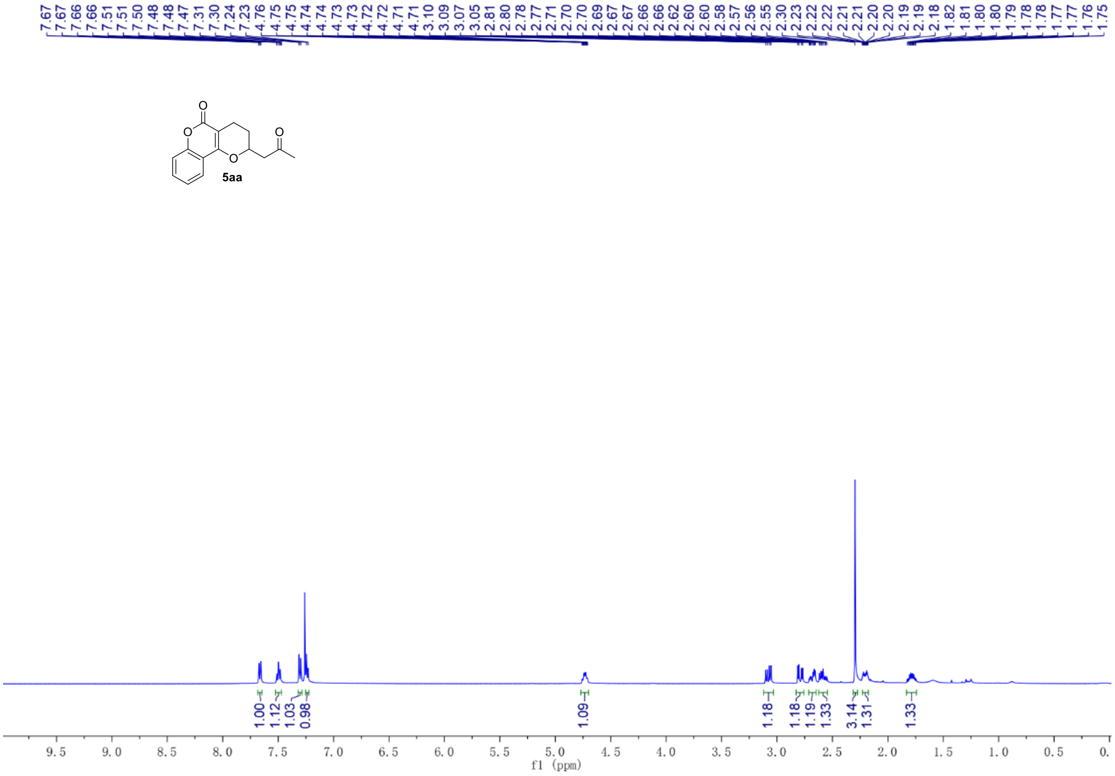

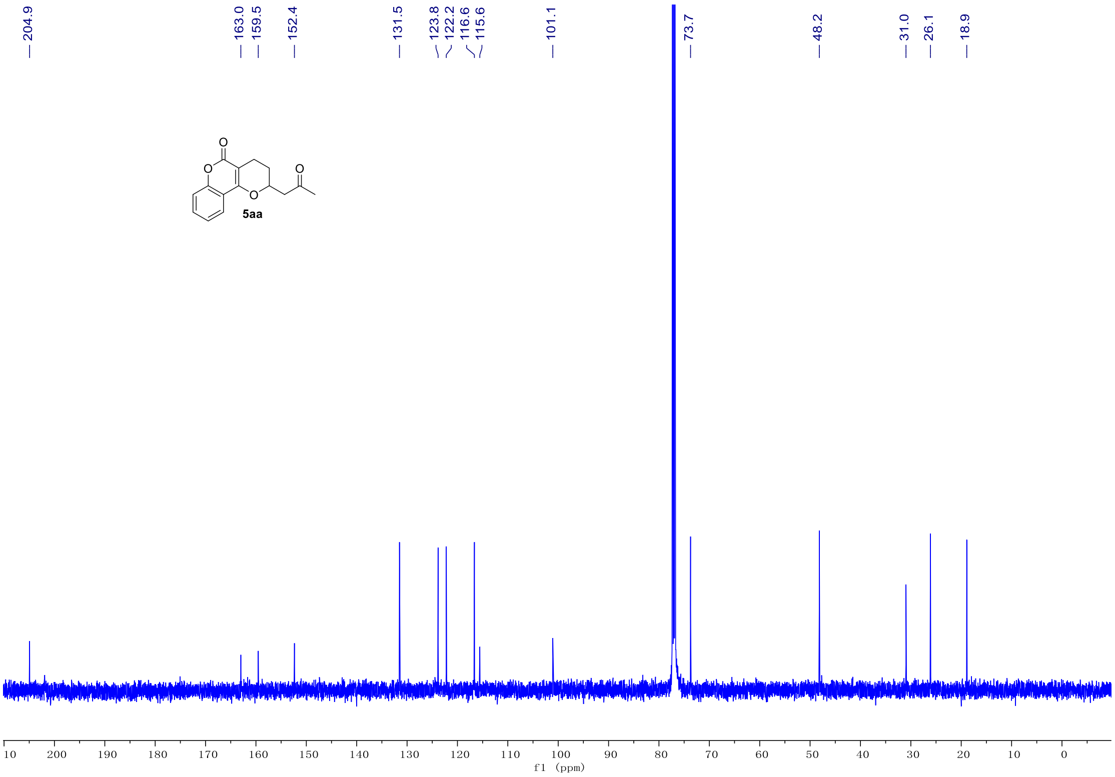

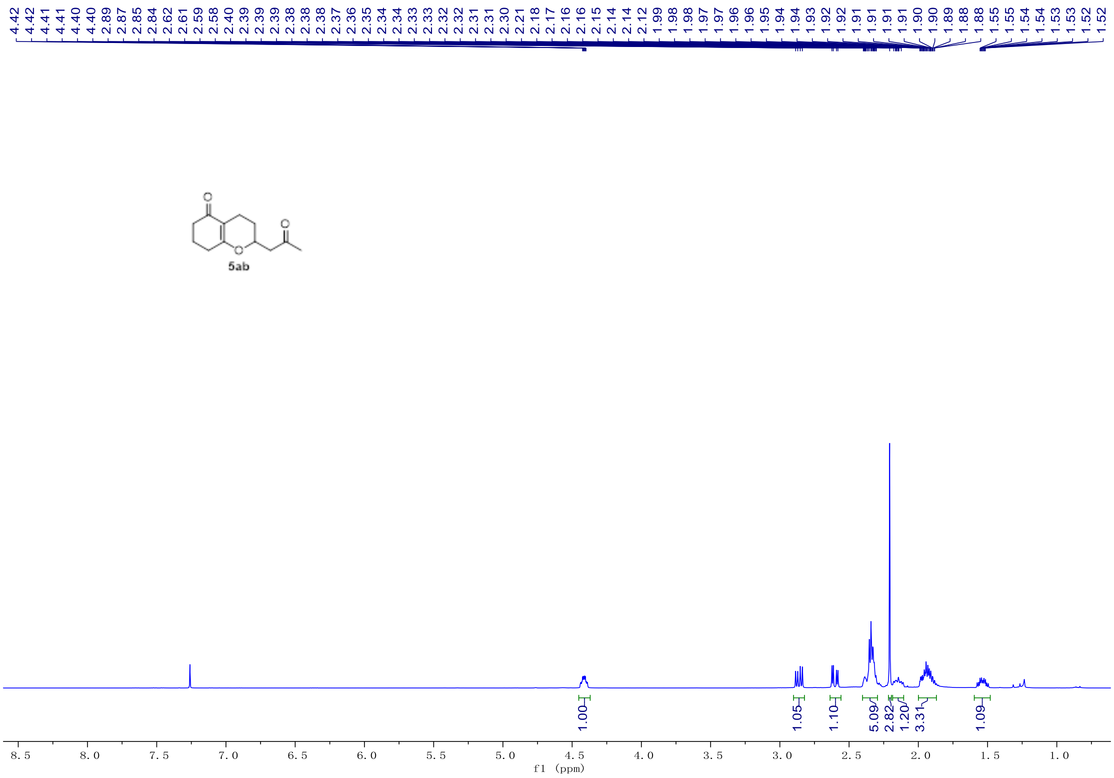
4.42 4.42 4.41 4.41 4.40 4.40 2.89 2.87 2.85 2.84 2.62 2.61 2.59 2.58 2.40 2.39 2.39 2.39 2.39 2.38 2.38 2.38 2.37 2.36 2.35 2.34 2.34 2.33 2.33 2.32 2.32 2.31 2.31 2.30 2.21 2.18 2.17 2.16 2.16 2.15 2.14 2.14 2.12 1.99 1.98 1.98 1.97 1.97 1.96 1.96 1.95 1.94 1.94 1.93 1.92 1.92 1.91 1.91 1.91 1.91 1.90 1.90 1.89 1.88 1.88 1.55 1.55 1.54 1.54 1.53 1.53 1.52 1.52
5ab
1.00
1.05
1.10
5.09
2.82
1.20
3.31
1.09
8.5 8.0 7.5 7.0 6.5 6.0 5.5 5.0 4.5 4.0 3.5 3.0 2.5 2.0 1.5 1.0
f1 (ppm)

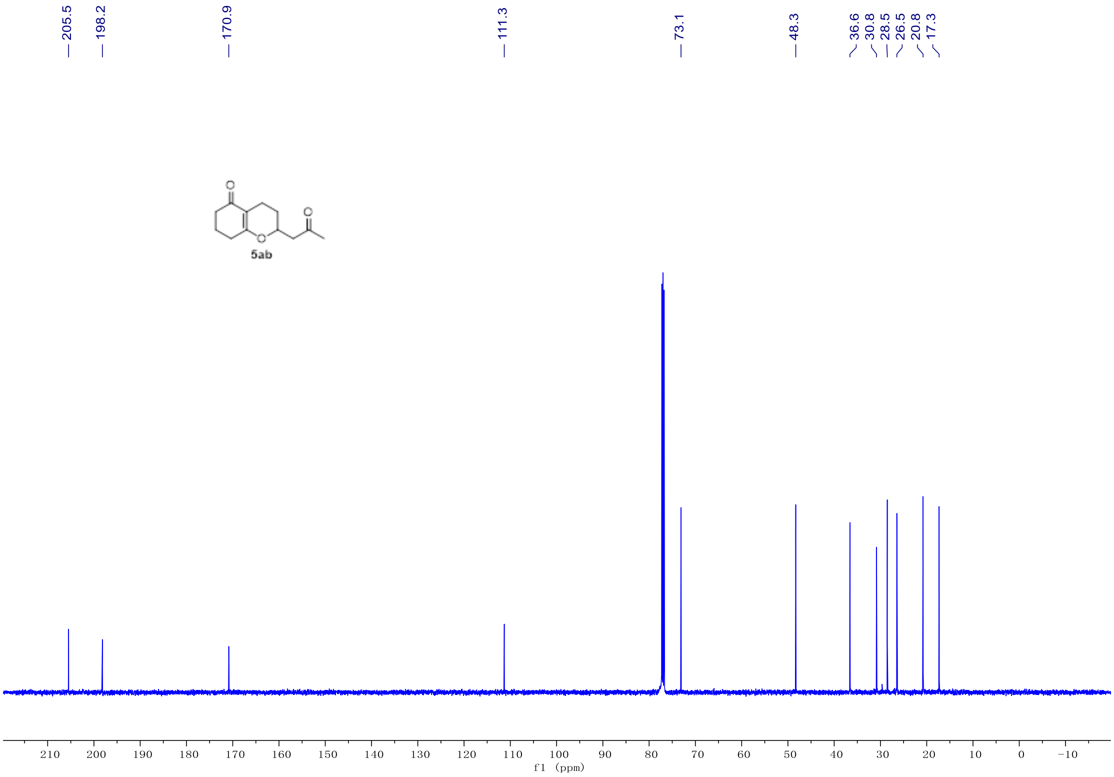
205.5
198.2
170.9
111.3
73.1
48.3
36.6 30.8 28.5 26.5 20.8 17.3
5ab
210 200 190 180 170 160 150 140 130 120 110 100 90 80 70 60 50 40 30 20 10 0 -10
f1 (ppm)

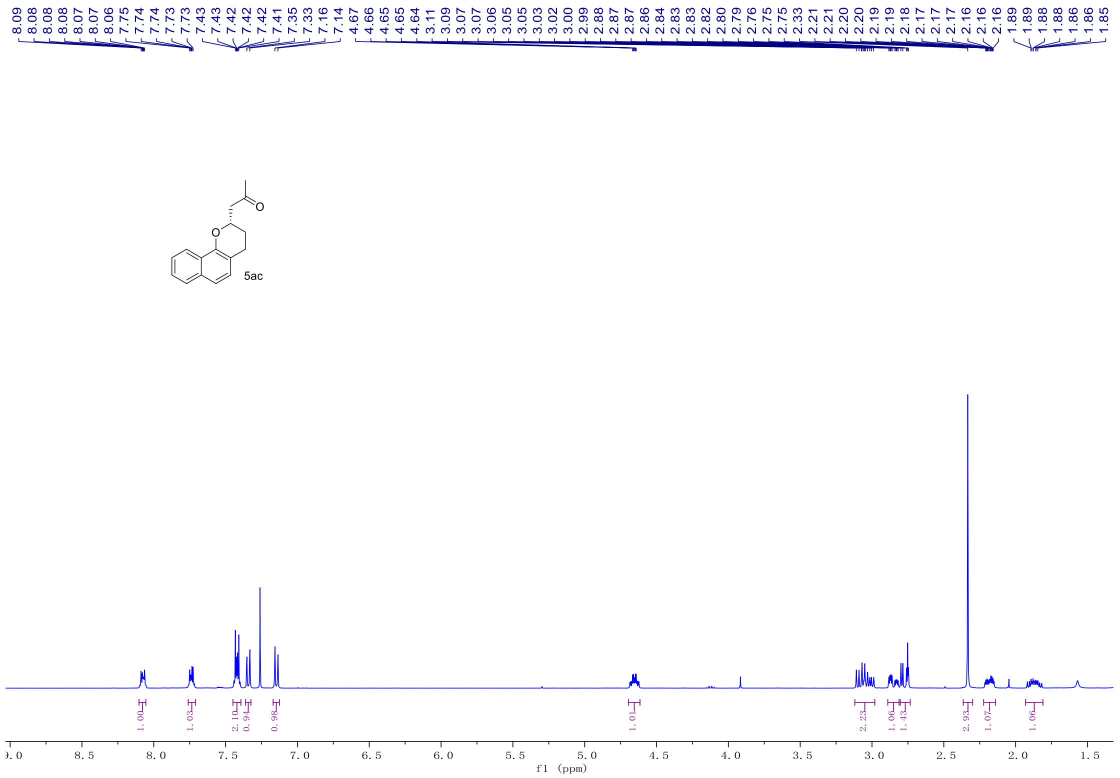
8.09 8.08 8.08 8.08 8.07 8.07 8.06 7.75 7.74 7.74 7.73 7.73 7.43 7.43 7.42 7.42 7.42 7.41 7.35 7.33 7.16 7.14 4.67 4.66 4.65 4.65 4.64 3.11 3.09 3.07 3.07 3.06 3.05 3.05 3.03 3.02 3.00 2.99 2.88 2.87 2.87 2.86 2.84 2.83 2.83 2.82 2.80 2.79 2.76 2.75 2.75 2.33 2.21 2.21 2.20 2.20 2.19 2.19 2.18 2.17 2.17 2.17 2.16 2.16 2.16 1.89 1.89 1.88 1.88 1.86 1.85
O
O
5ac
1.00 1.03 2.10 0.94 0.98 1.01 2.23 1.06 1.43 2.93 1.07 1.06
f1 (ppm)

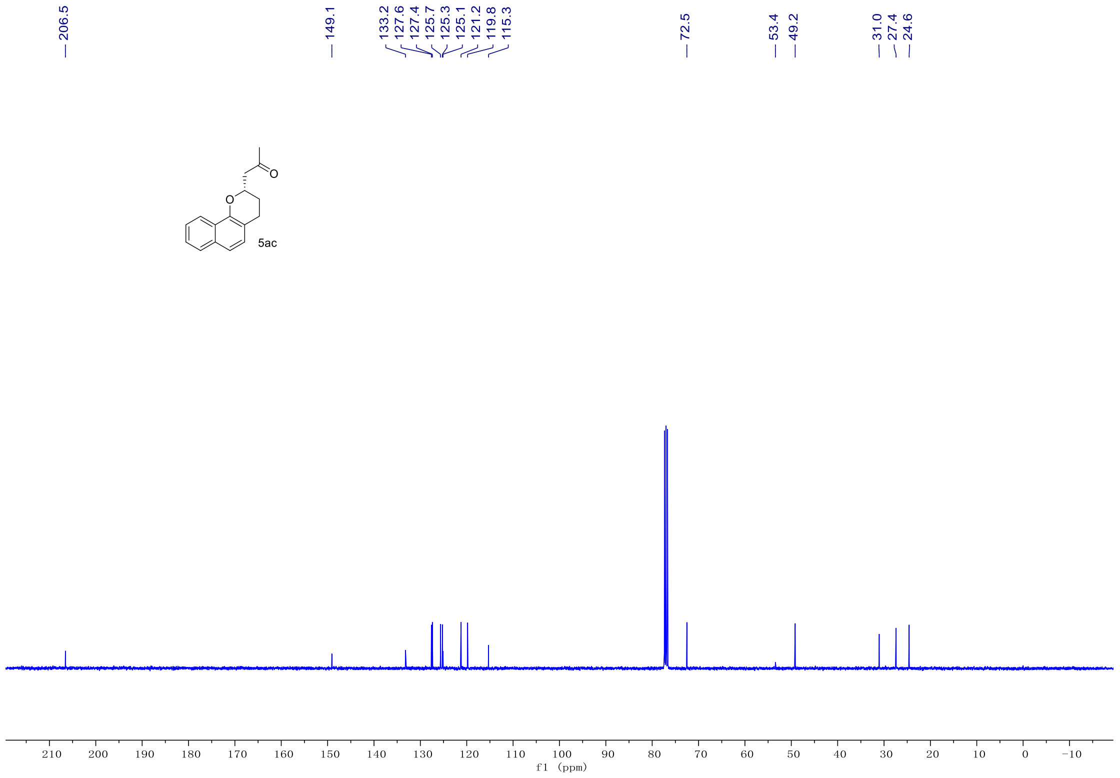
206.5 149.1 133.2 127.6 127.4 125.7 125.3 125.1 121.2 119.8 115.3 72.5 53.4 49.2 31.0 27.4 24.6
O
O
5ac
f1 (ppm)

4. Copies of HRMS Spectra for Compounds

Elemental Composition Report **Page 1**

Single Mass Analysis
Tolerance = 50.0 PPM / DBE: min = -1.5, max = 50.0
Element prediction: Off
Number of isotope peaks used for i-FIT = 3

Monoisotopic Mass, Even Electron Ions
194 formula(e) evaluated with 1 results within limits (all results (up to 1000) for each mass)
Elements Used:
C: 16-16 H: 0-41 N: 0-8 O: 0-50 Se: 0-2 Na: 1-1

8-
230308-2-310-2-4AA 9 (0.118) 1: TOF MS ES+ 1.51e+004

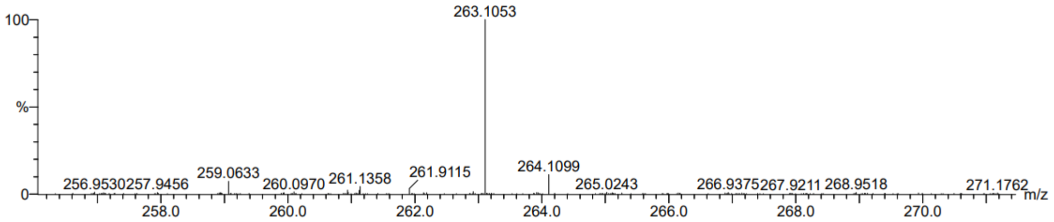

Minimum:				-1.5				
Maximum:		5.0	50.0	50.0				
Mass	Calc. Mass	mDa	PPM	DBE	i-FIT	Norm	Conf(%)	Formula
263.1053	263.1048	0.5	1.9	8.5	266.7	n/a	n/a	C16 H16 O2 Na

The HRMS Spectra of **4aa**

Elemental Composition Report **Page 1**

Single Mass Analysis
Tolerance = 20.0 PPM / DBE: min = -1.5, max = 50.0
Element prediction: Off
Number of isotope peaks used for i-FIT = 3

Monoisotopic Mass, Even Electron Ions
167 formula(e) evaluated with 1 results within limits (up to 50 closest results for each mass)
Elements Used:
C: 18-18 H: 19-19 N: 0-100 O: 0-100
2
230706-5-388-1-4AB 6 (0.085) 1: TOF MS ES+ 8.66e+005

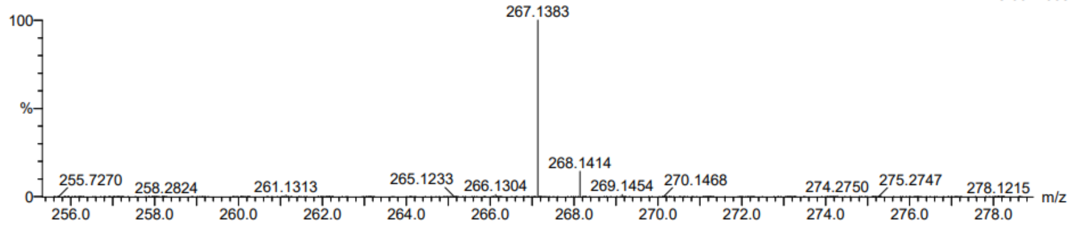

Minimum:				-1.5				
Maximum:		5.0	20.0	50.0				
Mass	Calc. Mass	mDa	PPM	DBE	i-FIT	Norm	Conf(%)	Formula
267.1383	267.1385	-0.2	-0.7	9.5	250.8	n/a	n/a	C18 H19 O2

The HRMS Spectra of **4ab**

Elemental Composition Report

Page 1

Single Mass Analysis

Tolerance = 20.0 PPM / DBE: min = -1.5, max = 50.0
Element prediction: Off
Number of isotope peaks used for i-FIT = 3

Monoisotopic Mass, Even Electron Ions
216 formula(e) evaluated with 1 results within limits (up to 50 closest results for each mass)
Elements Used:
C: 21-21 H: 19-19 N: 0-100 O: 0-100
2
230706-5-388-1-4AC 8 (0.102)   1: TOF MS ES+ 1.29e+005

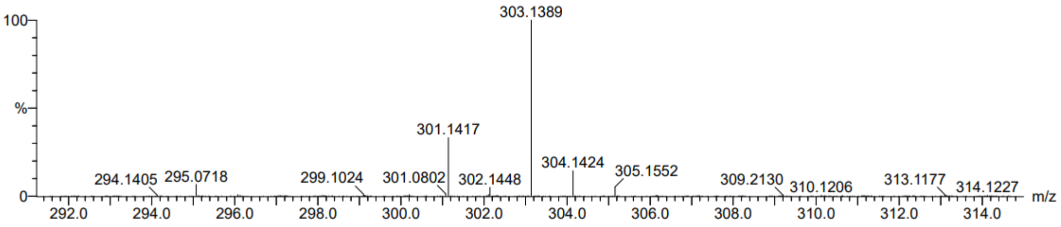

Minimum:				-1.5				
Maximum:		5.0	20.0	50.0				

Mass	Calc. Mass	mDa	PPM	DBE	i-FIT	Norm	Conf(%)	Formula
303.1389	303.1385	0.4	1.3	12.5	319.1	n/a	n/a	C21 H19 O2

The HRMS Spectra of **4ac**

Elemental Composition Report

Page 1

Single Mass Analysis

Tolerance = 20.0 PPM / DBE: min = -1.5, max = 50.0
Element prediction: Off
Number of isotope peaks used for i-FIT = 3

Monoisotopic Mass, Even Electron Ions
253 formula(e) evaluated with 1 results within limits (up to 50 closest results for each mass)
Elements Used:
C: 22-22 H: 21-21 N: 0-100 O: 0-100
2
230706-5-388-1-4AD 7 (0.093)   1: TOF MS ES+ 4.36e+006

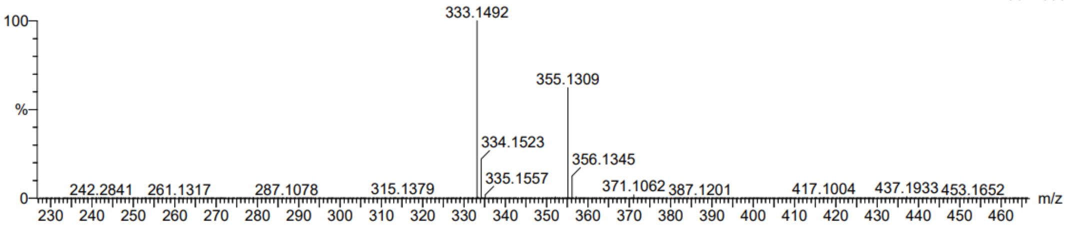

Minimum:				-1.5				
Maximum:		5.0	20.0	50.0				

Mass	Calc. Mass	mDa	PPM	DBE	i-FIT	Norm	Conf(%)	Formula
333.1492	333.1491	0.1	0.3	12.5	395.8	n/a	n/a	C22 H21 O3

The HRMS Spectra of **4ad**

Elemental Composition Report **Page 1**

Single Mass Analysis
Tolerance = 20.0 PPM / DBE: min = -1.5, max = 50.0
Element prediction: Off
Number of isotope peaks used for i-FIT = 3

Monoisotopic Mass, Even Electron Ions
253 formula(e) evaluated with 1 results within limits (up to 50 closest results for each mass)
Elements Used:
C: 22-22 H: 21-21 N: 0-100 O: 0-100
2
230706-5-388-1-4AE 8 (0.102) 1: TOF MS ES+ 1.42e+006

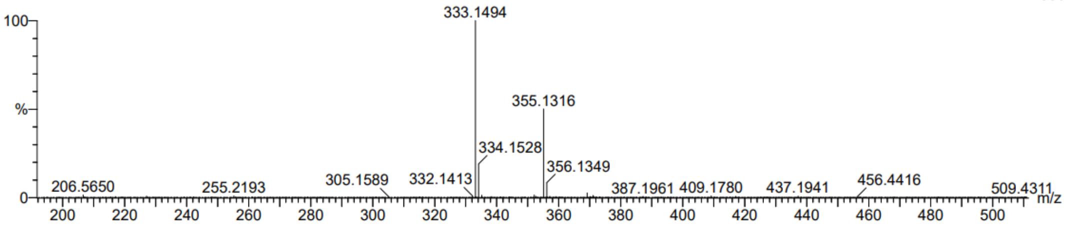

Minimum:				-1.5				
Maximum:		5.0	20.0	50.0				
Mass	Calc. Mass	mDa	PPM	DBE	i-FIT	Norm	Conf(%)	Formula
333.1494	333.1491	0.3	0.9	12.5	345.7	n/a	n/a	C22 H21 O3

The HRMS Spectra of **4ae**

Elemental Composition Report **Page 1**

Single Mass Analysis
Tolerance = 20.0 PPM / DBE: min = -1.5, max = 50.0
Element prediction: Off
Number of isotope peaks used for i-FIT = 3

Monoisotopic Mass, Even Electron Ions
937 formula(e) evaluated with 1 results within limits (up to 50 closest results for each mass)
Elements Used:
C: 22-22 H: 20-20 N: 0-20 O: 0-30 Na: 0-3
14
230303-1-307-1-4af 6 (0.085) 1: TOF MS ES+ 2.60e+006

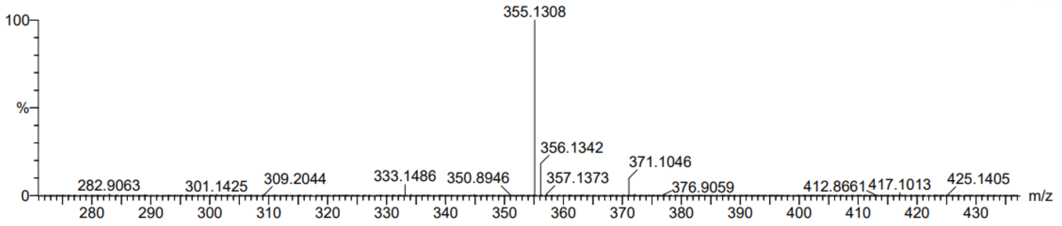

Minimum:				-1.5				
Maximum:		5.0	20.0	50.0				
Mass	Calc. Mass	mDa	PPM	DBE	i-FIT	Norm	Conf(%)	Formula
355.1308	355.1310	-0.2	-0.6	12.5	299.3	n/a	n/a	C22 H20 O3 Na

The HRMS Spectra of **4af**

Elemental Composition Report

Page 1

Single Mass Analysis

Tolerance = 20.0 PPM / DBE: min = -1.5, max = 50.0
Element prediction: Off
Number of isotope peaks used for i-FIT = 3

Monoisotopic Mass, Even Electron Ions
235 formula(e) evaluated with 1 results within limits (up to 50 closest results for each mass)
Elements Used:
C: 22-22 H: 21-21 N: 0-100 O: 0-100

2
230706-5-388-1-4AG 8 (0.102) 1: TOF MS ES+ 4.22e+005

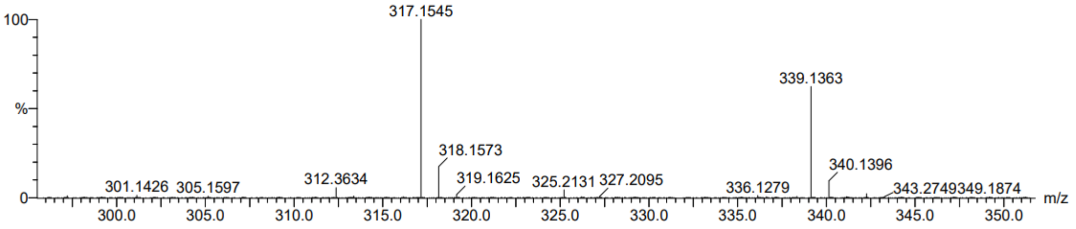

Minimum:				-1.5				
Maximum:		5.0	20.0	50.0				
Mass	Calc. Mass	mDa	PPM	DBE	i-FIT	Norm	Conf(%)	Formula
317.1545	317.1542	0.3	0.9	12.5	404.9	n/a	n/a	C22 H21 O2

The HRMS Spectra of **4ag**

Elemental Composition Report

Page 1

Single Mass Analysis

Tolerance = 20.0 PPM / DBE: min = -1.5, max = 50.0
Element prediction: Off
Number of isotope peaks used for i-FIT = 3

Monoisotopic Mass, Even Electron Ions
1257 formula(e) evaluated with 1 results within limits (up to 50 closest results for each mass)
Elements Used:
C: 21-21 H: 17-17 N: 0-20 O: 0-30 Na: 0-3 Br: 1-2

14
230303-1-307-1-4ai 7 (0.093) 1: TOF MS ES+ 1.06e+006

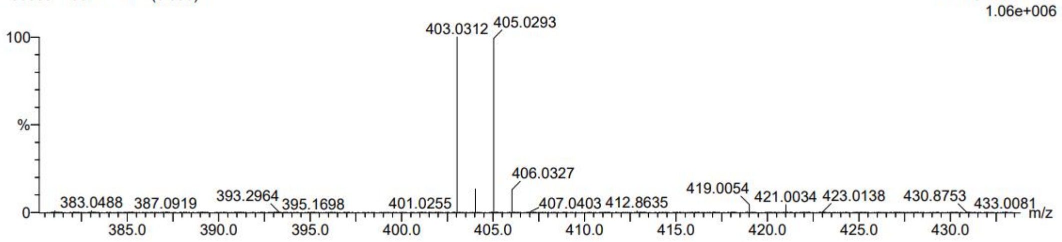

Minimum:				-1.5				
Maximum:		5.0	20.0	50.0				
Mass	Calc. Mass	mDa	PPM	DBE	i-FIT	Norm	Conf(%)	Formula
403.0312	403.0310	0.2	0.5	12.5	436.7	n/a	n/a	C21 H17 O2 Na Br

The HRMS Spectra of **4ah**

Elemental Composition Report

Page 1

Single Mass Analysis

Tolerance = 20.0 PPM / DBE: min = -1.5, max = 50.0
Element prediction: Off
Number of isotope peaks used for i-FIT = 3

Monoisotopic Mass, Even Electron Ions
1940 formula(e) evaluated with 1 results within limits (up to 50 closest results for each mass)
Elements Used:
C: 21-21 H: 17-17 N: 0-20 O: 0-30 Na: 0-3 Cl: 1-3
14
230303-1-307-1-4ah 8 (0.102)

1: TOF MS ES+
4.68e+004

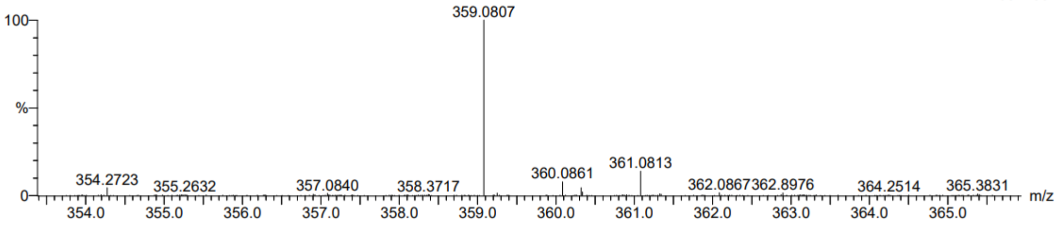

Minimum:				-1.5				
Maximum:		5.0	20.0	50.0				
Mass	Calc. Mass	mDa	PPM	DBE	i-FIT	Norm	Conf(%)	Formula
359.0807	359.0815	-0.8	-2.2	12.5	362.4	n/a	n/a	C21 H17 O2 Na Cl

The HRMS Spectra of **4ai**

Elemental Composition Report

Page 1

Single Mass Analysis

Tolerance = 20.0 PPM / DBE: min = -1.5, max = 50.0
Element prediction: Off
Number of isotope peaks used for i-FIT = 3

Monoisotopic Mass, Even Electron Ions
562 formula(e) evaluated with 1 results within limits (up to 50 closest results for each mass)
Elements Used:
C: 16-16 H: 16-16 N: 0-20 O: 0-30 Na: 0-3
14
230303-1-307-1-4aj 8 (0.102)

1: TOF MS ES+
2.62e+006

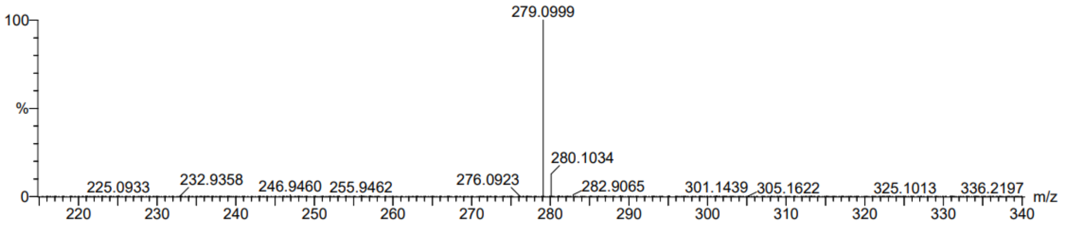

Minimum:				-1.5				
Maximum:		5.0	20.0	50.0				
Mass	Calc. Mass	mDa	PPM	DBE	i-FIT	Norm	Conf(%)	Formula
279.0999	279.0997	0.2	0.7	8.5	370.2	n/a	n/a	C16 H16 O3 Na

The HRMS Spectra of **4aj**

Elemental Composition Report

Single Mass Analysis
Tolerance = 20.0 PPM / DBE: min = -1.5, max = 50.0
Element prediction: Off
Number of isotope peaks used for i-FIT = 3

Monoisotopic Mass, Even Electron Ions
625 formula(e) evaluated with 1 results within limits (up to 50 closest results for each mass)
Elements Used:
C: 17-17 H: 18-18 N: 0-20 O: 0-30 Na: 0-3
14
230303-1-307-1-4ak 8 (0.102) 1: TOF MS ES+ 2.27e+006

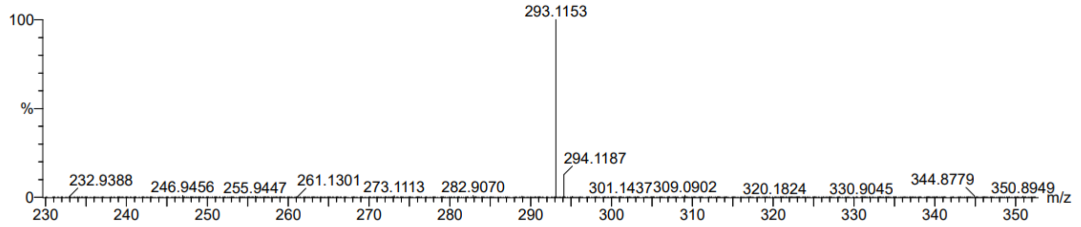

Minimum:				-1.5
Maximum:		5.0	20.0	50.0

Mass	Calc. Mass	mDa	PPM	DBE	i-FIT	Norm	Conf(%)	Formula
293.1153	293.1154	-0.1	-0.3	8.5	335.8	n/a	n/a	C17 H18 O3 Na

The HRMS Spectra of **4ak**

Elemental Composition Report

Page 1

Single Mass Analysis
Tolerance = 20.0 PPM / DBE: min = -1.5, max = 50.0
Element prediction: Off
Number of isotope peaks used for i-FIT = 3

Monoisotopic Mass, Even Electron Ions
208 formula(e) evaluated with 1 results within limits (up to 50 closest results for each mass)
Elements Used:
C: 16-16 H: 16-16 N: 0-100 O: 0-100 Br: 1-2

2
230706-5-388-1-4BA 7 (0.093) 1: TOF MS ES+ 1.59e+003

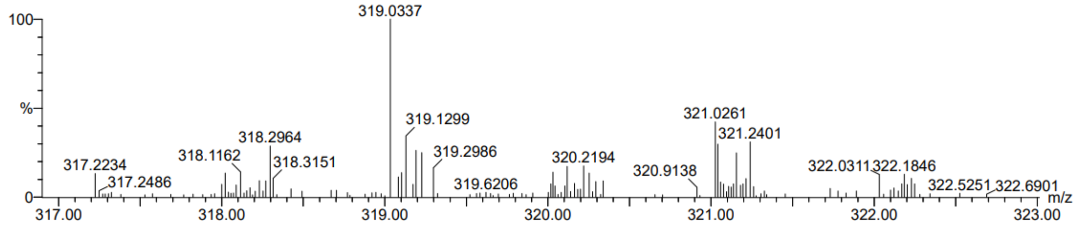

Minimum:				-1.5
Maximum:		5.0	20.0	50.0

Mass	Calc. Mass	mDa	PPM	DBE	i-FIT	Norm	Conf(%)	Formula
319.0337	319.0334	0.3	0.9	8.5	526.4	n/a	n/a	C16 H16 O2 Br

The HRMS Spectra of **4ba**

Elemental Composition Report **Page 1**

Single Mass Analysis
Tolerance = 20.0 PPM / DBE: min = -1.5, max = 50.0
Element prediction: Off
Number of isotope peaks used for i-FIT = 3

Monoisotopic Mass, Even Electron Ions
208 formula(e) evaluated with 1 results within limits (up to 50 closest results for each mass)
Elements Used:
C: 16-16 H: 16-16 N: 0-100 O: 0-100 Br: 1-2

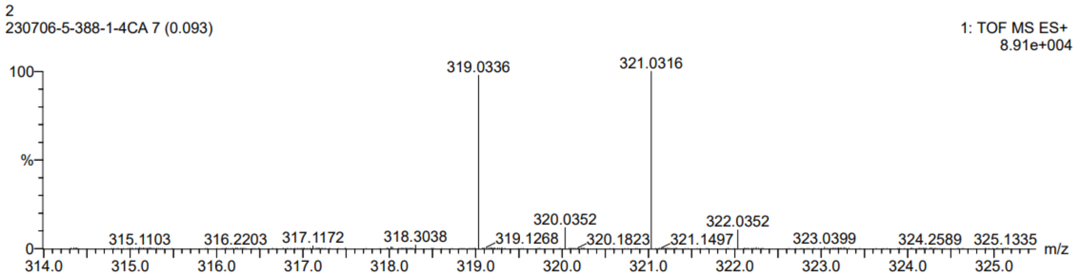

Minimum:				-1.5				
Maximum:		5.0	20.0	50.0				
Mass	Calc. Mass	mDa	PPM	DBE	i-FIT	Norm	Conf(%)	Formula
319.0336	319.0334	0.2	0.6	8.5	443.7	n/a	n/a	C16 H16 O2 Br

The HRMS Spectra of **4ca**

Elemental Composition Report **Page 1**

Single Mass Analysis
Tolerance = 20.0 PPM / DBE: min = -1.5, max = 50.0
Element prediction: Off
Number of isotope peaks used for i-FIT = 3

Monoisotopic Mass, Even Electron Ions
527 formula(e) evaluated with 1 results within limits (up to 50 closest results for each mass)
Elements Used:
C: 17-17 H: 16-16 N: 0-20 O: 0-30 Na: 0-3

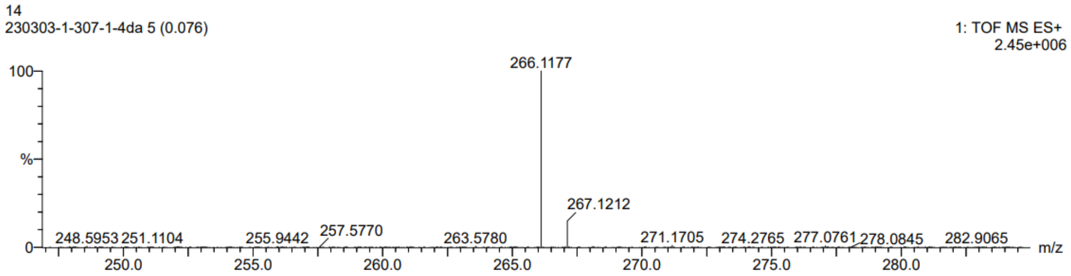

Minimum:				-1.5				
Maximum:		5.0	20.0	50.0				
Mass	Calc. Mass	mDa	PPM	DBE	i-FIT	Norm	Conf(%)	Formula
266.1177	266.1181	-0.4	-1.5	10.5	379.4	n/a	n/a	C17 H16 N O2

The HRMS Spectra of **4da**

Elemental Composition Report

Page 1

Single Mass Analysis

Tolerance = 20.0 PPM / DBE: min = -1.5, max = 50.0
Element prediction: Off
Number of isotope peaks used for i-FIT = 3

Monoisotopic Mass, Even Electron Ions
123 formula(e) evaluated with 1 results within limits (up to 50 closest results for each mass)
Elements Used:
C: 13-13 H: 15-15 N: 0-100 O: 0-100
2
230706-5-388-1-4EA 5 (0.076)

1: TOF MS ES+
2.69e+005

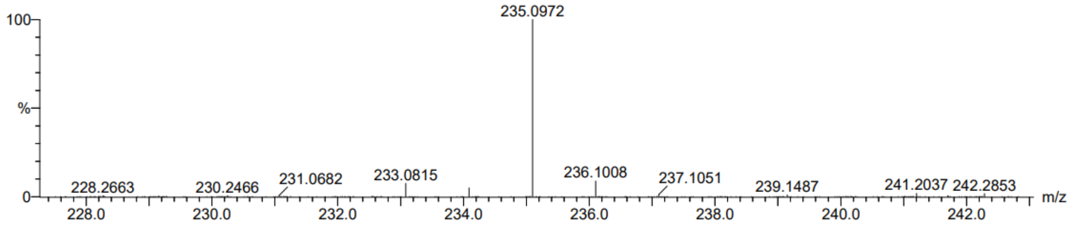

Minimum:				-1.5				
Maximum:		5.0	20.0	50.0				

Mass	Calc. Mass	mDa	PPM	DBE	i-FIT	Norm	Conf(%)	Formula
235.0972	235.0970	0.2	0.9	6.5	315.4	n/a	n/a	C13 H15 O4

The HRMS Spectra of **4ea**

Elemental Composition Report

Page 1

Single Mass Analysis

Tolerance = 20.0 PPM / DBE: min = -1.5, max = 50.0
Element prediction: Off
Number of isotope peaks used for i-FIT = 3

Monoisotopic Mass, Even Electron Ions
168 formula(e) evaluated with 1 results within limits (up to 50 closest results for each mass)
Elements Used:
C: 17-17 H: 19-19 N: 0-100 O: 0-100
6
230710-5-391-4fa 7 (0.093)

1: TOF MS ES+
7.06e+005

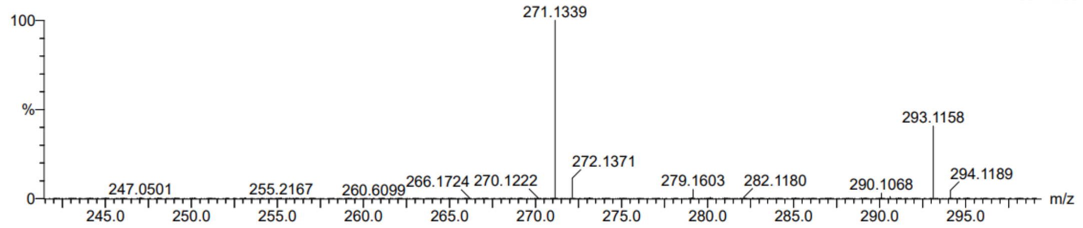

Minimum:				-1.5				
Maximum:		5.0	20.0	50.0				

Mass	Calc. Mass	mDa	PPM	DBE	i-FIT	Norm	Conf(%)	Formula
271.1339	271.1334	0.5	1.8	8.5	555.1	n/a	n/a	C17 H19 O3

The HRMS Spectra of **4fa**

Elemental Composition Report

Single Mass Analysis

Tolerance = 20.0 PPM / DBE: min = -1.5, max = 50.0
Element prediction: Off
Number of isotope peaks used for i-FIT = 3

Monoisotopic Mass, Even Electron Ions
625 formula(e) evaluated with 1 results within limits (up to 50 closest results for each mass)
Elements Used:
C: 17-17 H: 18-18 N: 0-20 O: 0-30 Na: 0-3
14
230303-1-307-1-4ja 8 (0.102)

1: TOF MS ES+
2.73e+006

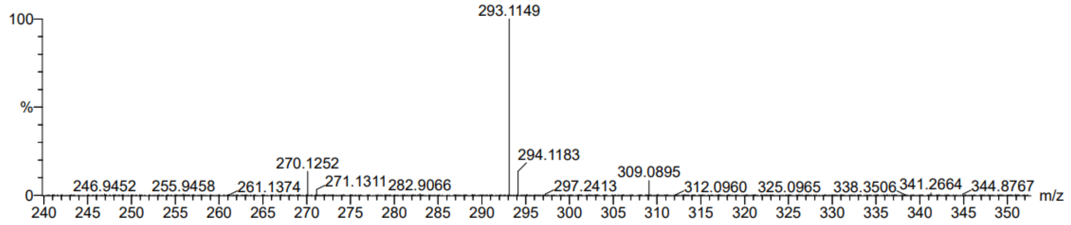

Minimum:				-1.5				
Maximum:		5.0	20.0	50.0				

Mass	Calc. Mass	mDa	PPM	DBE	i-FIT	Norm	Conf(%)	Formula
293.1149	293.1154	-0.5	-1.7	8.5	319.3	n/a	n/a	C17 H18 O3 Na

The HRMS Spectra of **4ga**

Elemental Composition Report

Page 1

Single Mass Analysis

Tolerance = 50.0 PPM / DBE: min = -1.5, max = 50.0
Element prediction: Off
Number of isotope peaks used for i-FIT = 3

Monoisotopic Mass, Even Electron Ions
219 formula(e) evaluated with 1 results within limits (all results (up to 1000) for each mass)
Elements Used:
C: 15-15 H: 0-41 N: 0-8 O: 0-50 Na: 1-1 Se: 0-2
8-
230308-2-310-2-4HA 5 (0.076)

1: TOF MS ES+
1.84e+004

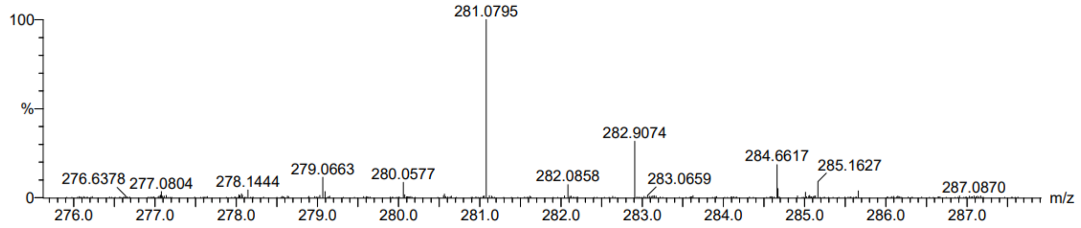

Minimum:				-1.5				
Maximum:		5.0	50.0	50.0				

Mass	Calc. Mass	mDa	PPM	DBE	i-FIT	Norm	Conf(%)	Formula
281.0795	281.0790	0.5	1.8	8.5	363.7	n/a	n/a	C15 H14 O4 Na

The HRMS Spectra of **5aa**

Elemental Composition Report **Page 1**

Single Mass Analysis
Tolerance = 20.0 PPM / DBE: min = -1.5, max = 50.0
Element prediction: Off
Number of isotope peaks used for i-FIT = 3

Monoisotopic Mass, Even Electron Ions
363 formula(e) evaluated with 1 results within limits (up to 50 closest results for each mass)
Elements Used:
C: 12-12 H: 16-16 N: 0-20 O: 0-30 Na: 0-3

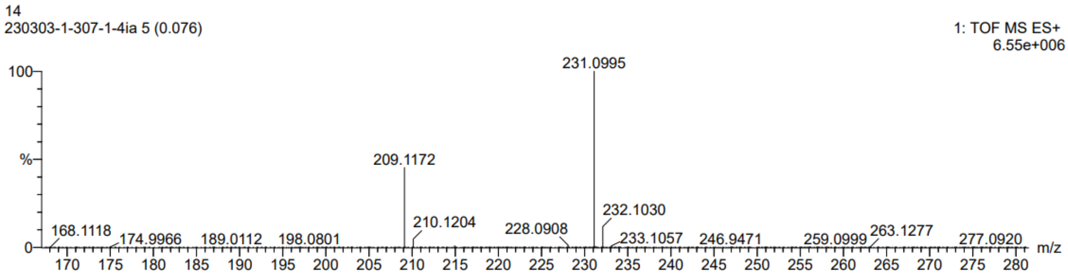

Minimum:				-1.5				
Maximum:		5.0	20.0	50.0				
Mass	Calc. Mass	mDa	PPM	DBE	i-FIT	Norm	Conf(%)	Formula
231.0995	231.0997	-0.2	-0.9	4.5	469.4	n/a	n/a	C12 H16 O3 Na

The HRMS Spectra of **5ab**
